# Syntactic flexibility and lexical encoding in aging sentence production: an eye tracking study

**DOI:** 10.3389/fpsyg.2024.1304517

**Published:** 2024-08-26

**Authors:** Joshua D. Weirick, Jiyeon Lee

**Affiliations:** Aphasia Research Laboratory, Department of Speech, Language, and Hearing Sciences, Purdue University, West Lafayette, IN, United States

**Keywords:** lexical processing, priming, healthy aging, sentence production, eye tracking

## Abstract

**Purpose:**

Successful sentence production requires lexical encoding and ordering them into a correct syntactic structure. It remains unclear how different processes involved in sentence production are affected by healthy aging. We investigated (a) if and how aging affects lexical encoding and syntactic formulation during sentence production, using auditory lexical priming and eye tracking-while-speaking paradigms and (b) if and how verbal working memory contributes to age-related changes in sentence production.

**Methods:**

Twenty older and 20 younger adults described transitive and dative action pictures following auditory lexical primes, by which the relative ease of encoding the agent or theme nouns (for transitive pictures) and the theme and goal nouns (for dative pictures) was manipulated. The effects of lexical priming on off-line syntactic production and real-time eye fixations to the primed character were measured.

**Results:**

In offline production, older adults showed comparable priming effects to younger adults, using the syntactic structure that allows earlier mention of the primed lexical item in both transitive and dative sentences. However, older adults showed longer lexical priming effects on eye fixations to the primed character during the early stages of sentence planning. Preliminary analysis indicated that reduced verbal working memory may in part account for longer lexical encoding, particularly for older adults.

**Conclusion:**

These findings indicate that syntactic flexibility for formulating different grammatical structures remains largely robust with aging. However, lexical encoding processes are more susceptible to age-related changes, possibly due to changes in verbal working memory.

## 1 Introduction

Successful language production requires multiple cognitive-linguistic processes, including but not limited to encoding and retrieving individual lexical items and producing them into correct syntactic structures (Levelt, 1989; Bock and Levelt, 1994; Ferreira et al., 2018; Slevc, 2023). As the older adult population is growing at an unprecedented rate around the world (Roser, 2020), it becomes increasingly critical to study how healthy aging affects different processes of language production. It is well known that healthy aging does not affect all aspects of cognitive-linguistic processes (Peelle, 2019; Burke and Shafto, 2008). For example, lexical encoding and retrieval become less efficient with aging, resulting in frequent tip-of-the-tongue and word-finding errors in older adults (Burke et al., 1991; Shafto et al., 2007; Au et al., 1995; Connor et al., 2004; Feyereisen, 1997; Mortensen et al., 2006; Verhaegen and Poncelet, 2013). Some cognitive abilities, such as verbal working memory, decline with aging, adding challenges to various language production tasks (Waters and Caplan, 2003; Bopp and Verhaeghen, 2005). In contrast, certain aspects of language processing, such as vocabulary size and knowledge, continually increase with aging (Verhaeghen, 2003). However, the literature is less clear about how aging affects different processes involved in sentence production. The present study focuses on the impact of healthy aging on the lexical and syntactic processes involved in sentence production.

Studies focusing on sentence production show that older adults tend to produce fewer complex sentences, such as non-canonical sentences or left-branching structures in spoken narratives (Agmon et al., 2022; Kemper et al., 2001), sentence completion tasks (Kemper et al., 2004) and written diaries (Kemper, 1987). Reduced production of complex sentences in older adults is also found in more controlled tasks. In experiment 1 of Kemper et al.’s (2003) study, for example, the authors displayed two, three, and four words on the computer screen and asked the participants to construct sentences. The words disappeared upon the participants’ initiation of sentences, forcing them to remember the words to include in the sentences. Older adults, compared to young adults, produced more errors and shorter sentences when they had to use four words, but not with two and three words. Sung et al. (2024) using a picture description task, examined whether their Korean-speaking participants can flexibly choose to produce passive (the monkey is bitten by the dog) vs. active (the dog is biting the monkey) sentences after lexical priming of the theme or agent noun. Participants orally read either the theme (monkey) or the agent (dog) noun before they described the target action scene. Oral reading of the nouns served as a lexical prime by which the relative accessibility of respective nouns was manipulated, and it was expected that if the participants could generate syntactic structures flexibly, they would make more passive sentences than actives in the theme vs. agent prime condition. Both groups showed this pattern; however, older adults showed reduced production of passives after the theme prime compared to younger adults. Further, reduced production of passives in the theme prime condition was particularly true for older adults with lower verbal working memory, with no influence of working memory in younger adults, indicating working memory plays a more critical role during language processing in older adults (DeDe et al., 2014). Together, previous findings generally indicate that sentence production, especially for complex sentences, is vulnerable to age-related changes, as informed by the overall accuracy and frequency of target sentences, as well as the error patterns observed in non-target productions; however, they fail to reveal what specific aspects of sentence production might be impaired in aging.

In fact, studies show that when demands for non-syntactic processes are minimized, older adults do not always show a decline in complex sentence production. For example, in Davidson et al. (2003), the participants were asked to construct a set of transitive and dative sentences using written words. However, different from Kemper et al. (2003), their word stimuli were presented on the screen throughout sentence production, thus minimizing demands for lexical encoding and memory for words. Their older adults showed comparable production accuracy and latency times as young adults, indicating intact syntactic processing in aging. Studies using implicit syntactic priming generally show that older adults show robust ability to re-use previously encountered (primed) sentence structure in their subsequent sentence production, indicating preserved activation of syntactic representations (Hardy et al., 2017; Hardy et al., 2020; Lee et al., 2022). More recently, Hardy et al. (2020) specifically demonstrated that healthy aging disrupts lexical, but not syntactic, processes using speech initiation times of multi-word sentences (e.g., *the bell and the glove move up*) under syntactic priming and lexical preview manipulations. After being primed with a related syntactic structure (*the pig and the leaf move together*), older adults initiated their sentences as fast as young adults. When the participants previewed the object images, young adults showed speech preview benefits when they previewed both one (*bell*) and two images (*bell* and *glove*). However, older adults showed speed preview benefits for one noun but not for two, indicating that they have reduced lexical processing. Therefore, there is a need to more clearly delineate how different processes involved in sentence production, more specifically, the lexical and syntactic processes, are affected by healthy aging.

The current study aims to address this gap by systematically investigating whether and to what extent the processes of lexical encoding and syntactic structure formulation involved in the production of transitive and dative sentences are affected by healthy aging. We used an auditory lexical priming task in conjunction with eyetracking reported in Lee (2020) and similar to others (e.g., Sung et al., 2024; Slevc, 2011). The participants described target action-describing pictures (e.g., *a woman pulling a horse*) using sentences. Prior to target picture descriptions, the participants heard an auditory prompt that included a lexical prime target (e.g., *what’s happening with the horse?*). This lexical prime served to increase the relative ease of accessing and encoding the primed word (theme noun in this example) compared to the non-primed word (agent noun). Then, we measured if the participants would produce the syntactic structure that allows earlier mention of the primed word (e.g., *the horse is pulled by the woman* rather than *the woman is pulling the horse*) to assess their syntactic processing. We also measured if participants would gaze at the target character (*horse*) in the picture stimulus more quickly and to a greater extent in the primed vs. non-primed condition to assess real-time lexical encoding processes.

The current paradigm is well-suited to assess speakers’ ability to flexibility and efficiently generate syntactic structures within the theories of incremental language production. In incremental language production, speakers generate syntactic structures as individual lexical items are retrieved in a piece-by-piece manner (Bock and Ferreira, 2014; Bock and Levelt, 1994; Kempen and Hoenkamp, 1987; Levelt, 1989; Thompson et al., 2015). Therefore, the relative ease of accessing and encoding lexical information influences speakers’ choices of syntactic structures (Altmann and Kemper, 2006; Bock, 1986; Bock and Warren, 1985; Gleitman et al., 2007; Lee, 2020; Slevc, 2011). As exemplified above, when describing a transitive event, if the theme becomes more accessible, speakers are likely to produce the passive structure more frequently than they would otherwise (Lee, 2020; Sung et al., 2024). Similarly, for dative syntactic alternations, speakers are more likely to produce the double-object (DO) dative structure ‘the man gave the child a gift’ when the goal argument ‘child’ becomes more accessible than the theme ‘gift’, while the speaker is likely to produce the prepositional object (PO) dative structure ‘the man gave a gift to the child’ if the theme ‘gift’ becomes more accessible (Bock, 1986, 1987; Bock and Warren, 1985; Slevc, 2011).

Monitoring participants eye fixations to the target character in the picture scene following lexical priming allows an evaluation of speakers’ ability to access and encode lexical items in real-time. Previous eyetracking studies have established that difficulty encoding lexical items is closely correlated with how long speakers fixate on the target picture before naming (e.g., Meyer and Lethaus, 2004; Lee et al., 2015; Griffin, 2004). For example, speakers tend to show longer gaze durations for referents with low frequency names or those with low codability (*donkey/mule*) compared to those with high codability (*needle*) (Griffin, 2004; Lee et al., 2015; Lee, 2017). When producing sentences based on picture scenes, the time courses of eye fixations roughly align with different stages of early lexico-message encoding, sentence formation, and execution (Griffin and Bock, 2000; Bock et al., 2003; Bock and Ferreira, 2014; Henderson and Ferreira, 2013). The early fixations that happen from the picture onset through about 800 ms would be most revealing for our question, as studies show that during this early time window, speakers apprehend the visual scene to get a coarse encoding of the event structure and begin encoding lexical information for at least one of the characters in the scene to prepare to initiate their sentence (Griffin and Bock, 2000; Bock et al., 2003). After this phase, eye fixations to each character in the scene are often thought to reflect sentence formulation and speech execution, although much variability exists across studies using different tasks and strategies used by individual speakers (Griffin and Mouzon, 2004; Lee and Thompson, 2011a, 2011b; van de Velde et al., 2014; see also Henderson and Ferreira, 2013).

Lastly, we conducted an exploratory analysis to test if and how individuals’ verbal working memory may influence the off-line and real-time processes of sentence production in older and young adults. Verbal working memory is a capacity-limited cognitive system involved in allocating processing resources to multiple task demands (Just and Carpenter, 1992; Kane and Engle, 2000). Working memory capacity is often measured by various span (e.g., digit, word, reading, pointing) tasks or in a dual task (Bopp and Verhaeghen, 2005). Reduced working memory is commonly and consistently reported in older adults, using a wide range of working memory tasks (Bopp and Verhaeghen, 2005 for meta-analysis; Stine and Wingfield, 1987; Waters and Caplan, 2003). In addition, there is considerable evidence that individuals’ higher verbal working memory positively correlates with better comprehension of complex sentences in both young and older adults, with the individual differences associated with working memory being greater for older adults than young adults (e.g., Waters and Caplan, 2001; DeDe et al., 2004; Caplan et al., 2013; Caplan and Waters, 1999; Just and Carpenter, 1992; Sung et al., 2017).

Relatively little work has investigated the role of working memory in sentence production. Among studies that have, Slevc (2011) found that under a concurrent verbal memory load (delayed recall of two unrelated words), their young adults showed reduced ability to flexibly use dative alternating structures in response to an auditory lexical priming prompt (the same task used in the current study), compared to the no memory load condition. Further, Sung et al. (2024) found that individual participants’ verbal working memory scores obtained from a word span task significantly predicted the degree of syntactic flexibility in older adults but not in younger adults. That is, older adults who showed lower verbal working memory had a greater difficulty producing passive structures when the theme noun was primed, compared to the older adults with higher verbal working memory. These findings confirm that verbal working memory interacts with how speakers efficiently produce sentences, and such interaction might become more prominent for older adults as their working memory capacity changes across their lifespan. However, these studies have not yet clearly delineated why lower working memory leads to difficulties in sentence production owing to their use of offline task accuracy measures. Examining the contributions of working memory to real-time lexical and off-line syntactic production processes separately might shed light on this remaining question.

### 1.1 The current study

The current study investigated if and how syntactic and lexical processes involved in sentence production are affected by healthy aging. Using an auditory lexical priming paradigm (Lee, 2020; Slevc, 2011), we examined whether older and younger adults would flexibly produce the sentence structure that allows earlier mention of a primed word. During the task, we also recorded participants’ eye fixations to the target characters under primed and unprimed conditions to examine real-time lexical encoding during the early stages of sentence planning. In addition, a picture-pointing span task (DeDe et al., 2014) was administered to all participants, as described in the methods section, to assess their verbal working memory.

Three research questions were addressed in this study. The first question examined if older adults would show significant lexical priming effects in their choice of syntactic structure in offline sentence production, producing the word order that allows earlier mention of the primed word. Secondly, we investigated whether older adults would show lexical priming effects on eye fixations similar to those found in young adults. Lastly, we examined if and how individuals’ verbal working memory modulated different degrees of lexical priming effects in off-line production and eye fixation data. Based on previous literature on the effect of aging during sentence production and the possibility of a greater role of working memory in language processing in older adults than in young adults, our hypotheses were as follows: If aging affects lexical processing to a greater extent than syntactic processing, it was predicted that the group differences in real-time eye fixation data would be significant, while older adults would show comparable effects as young adults in offline syntactic production. If aging affects both syntactic and lexical processes, group differences in the lexical priming effects were expected in both offline production and eye fixations. Regarding our third, rather exploratory question, it was hypothesized that individuals’ picture pointing scores would show significant interactions with the degree of lexical priming in either off-line syntactic production or eye fixations to primed characters, or both, indicating the extent to which individual differences in working memory contribute to difficulties in specific processes involved in sentence production. We also hypothesized that such an interaction between working memory scores and priming effects might be significant in older adults but less so in younger adults.

## 2 Methods

### 2.1 Participants

Twenty older adults (8 females, 12 males; mean age = 71.2, range 62–82) and 20 younger adults (15 females, 5 males; mean age 24 years, range 19–32) were tested. The two groups were matched in years of education [older adults: *M* (SD) = 17.2 (2.7), range = 12–23; younger adults: *M* (SD) = 16.65 (2.2), range = 13–22]. The data are from a larger study focusing on sentence production in aphasia and healthy adults. Part of the older adults’ data was previously described in Lee (2020) to compare with data from speakers with aphasia. All participants passed a pure-tone hearing screening at 500, 1,000, and 2,000 Hz at 40 dB at least in one ear and reported normal or corrected-to-normal vision. All participants self-identified as monolingual speakers of North American English.

The participants’ test scores are presented in Table 1. As a screener for abnormal changes in cognitive-linguistic skills, the Cognitive-Linguistic Quick Test (CLQT, Helm-Estabrooks, 2001) was administered prior to study enrollment. The Composite Severity Rating (CSR) scores obtained across the domains of language, memory, attention, and executive functioning revealed that all participants scored within normal limits for their age on the CLQT. The Boston Naming Test (Kaplan et al., 1983) and the Shipley’s Vocabulary Test (SVT; Shipley and Burlingame, 1941) were used to assess participants’ expressive and receptive vocabulary, respectively. Scores on the Boston Naming Test did not differ significantly between groups (*t* = −1.07, df = 24.59, *p* = 0.29). However, older adults scored significantly higher on the Shipley Vocabulary Test compared to younger adults, as expected (*t* = 3.56, df = 28.25, *p* = 0.001). To assess participants’ verbal working memory, the picture-pointing span task (DeDe et al., 2014) was administered both forward and backward. Participants pointed to visually presented pictures that matched the words that were produced by the examiner. Across the trials, the number of words increased from 2 to 8. For the forward task, the participants pointed to the pictures in the order the words were presented, while for the backward task, the participants pointed to the pictures in the reversed order of their presentation. Scores were calculated by the number of correctly pointed pictures for both forward and backward tasks. Older adults scored significantly lower on the working memory forward score compared to younger adults (*t* = −2.89, df = 38, *p* = 0.006) but not on the working memory backward score (*t* = −1.71, df = 38, *p* = 0.10).

**Table 1:** Participants’ test scores.

	Older adults				Younger adults			
	Mean	*SD*	Min	Max	Mean	*SD*	Min	Max
CLQT CSR	3.9	0.04	3.8	4	4	0	4	4
BNT	55	5	46	60	57	2	52	60
SVT	36.3	5.7	27	57	31.2	2.9	26	35
WMF	114.5	18.4	104	160	130	17.2	104	160
WMB	106.5	15.7	88	138	114.8	14.9	72	134

CLQT CSR, Cognitive Linguistic-Quick Test Composite Severity Rating; BNT, Boston Naming Test; SVT, Shipley’s Vocabulary Test; WMF, working memory forward total score; WMB, working memory backward total score.

### 2.2 Materials and design

For target picture stimuli, we used a total of 18 transitive and 18 dative black-and-white line drawings (Lee, 2020). The full list of the target experimental stimuli is provided in the referenced OSF repository (see Data Availability Statement below). Transitive pictures featured events involving two animate characters (e.g., *a dog chasing a mailman*), two inanimate characters (e.g., *a truck towing a car*), or an inanimate agent and animate theme characters (e.g., *a ball hitting a boy*). For dative pictures, all stimuli featured events involving an animate agent argument, an inanimate theme argument, and an animate goal argument (e.g., *a boy giving a flower to a teacher*). Each target picture was tested twice across the two prime conditions, as exemplified in Table 2, for a total of 36 transitive and 36 dative trials per participant.

**Table 2:** Example stimuli for target pictures and auditory lexical prime prompts.

Target type	Prime type
Transitive target picture trials (*n* = 36) *‘a woman pulling a horse’*	Theme prime (*n* = 18) *What is happening with the **horse**?*
	Agent prime (*n* = 18) *What is happening with the **woman**?*
Dative target picture trials (*n* = 36) *‘a boy giving a flower to a teacher’*	Goal prime (*n* = 18) *What is happening with the boy and the **teacher**?*
	Theme prime (*n* = 18) *What is happening with the boy and the **flower**?*

To avoid testing the same picture twice within a session, we created two experimental session lists. Across the session lists, each picture was elicited only in one prime condition. For example, if a transitive picture was elicited in the agent prime condition in list 1, then the picture was elicited in the theme prime condition in list 2. Both transitive and dative target pictures were included in the list. All participants completed two sessions at least 2 weeks apart, completing one list per session. The order of the lists was counterbalanced across the participants to avoid stimulus presentation order effects. The presentation order of the stimuli within the list was randomized across the participants and sessions.

In addition, we prepared a total of 36 filler pictures of intransitive events (e.g., a ball bouncing, a man laughing) to intersperse across the transitive and dative target pictures within the experimental list. Fillers were included to minimize participants’ potential use of a certain production strategy (e.g., relying on one type of sentence structure). Each filler picture was repeated twice within the list to ensure that there are at least two filler pictures embedded between experimental (either transitive or dative) pictures. Thus, each experimental list consisted of 36 experimental items (18 transitive pictures, 18 dative pictures, and 72 filler pictures). The same filler pictures were used between the experimental lists.

### 2.3 Procedure

**Figure 1:**
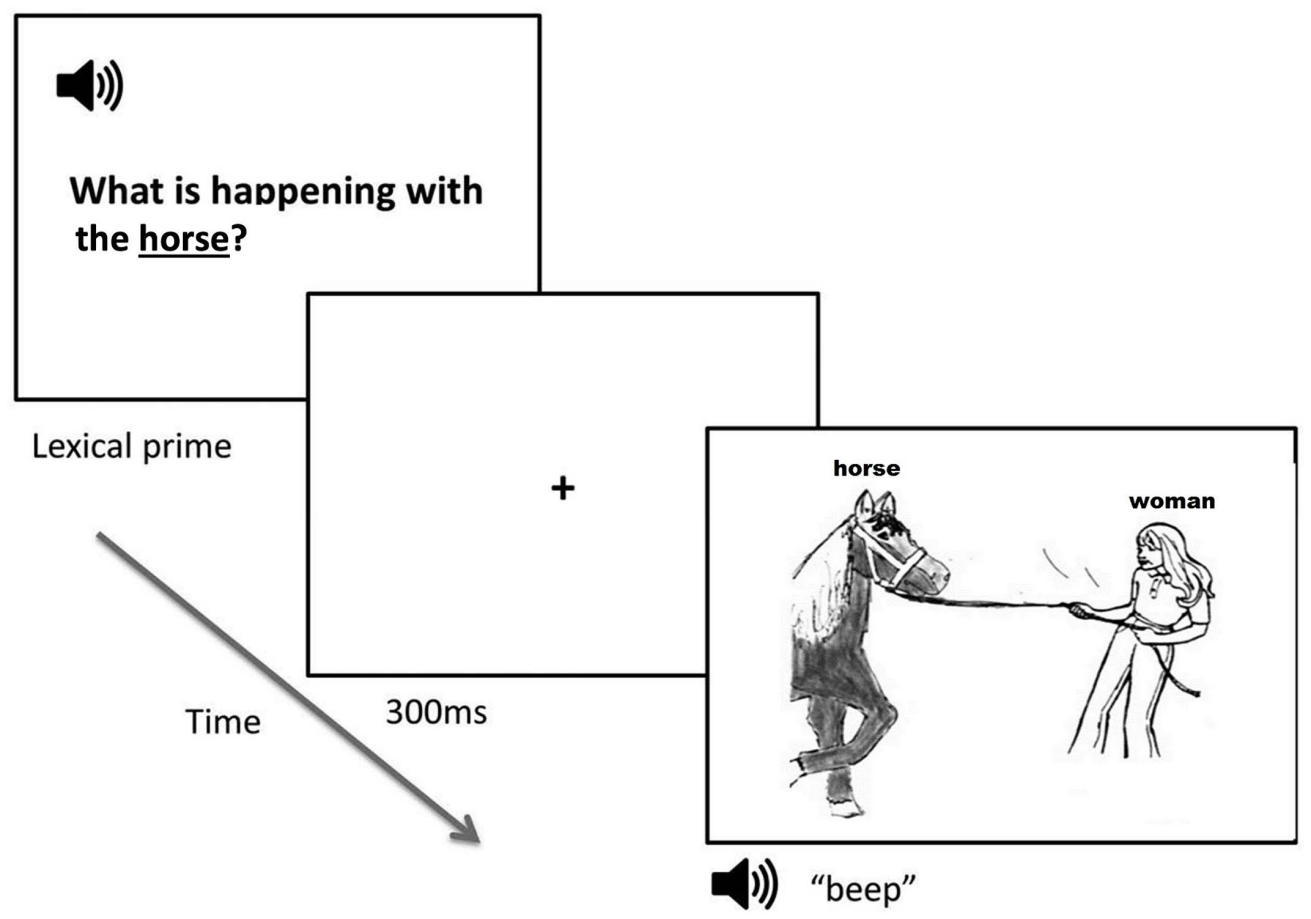
An example of a transitive experimental trial in the theme prime condition.

Participants completed a picture description task in conjunction with auditory lexical priming while their eye movements were tracked. The participant heard an auditory lexical prime prompt and then described a target picture using a sentence. The relative accessibility of different lexical items used in the target sentence was manipulated by varying the “givenness” of the nouns in the auditory prime (Slevc, 2011), as shown in Figure 1. For transitive targets (*a woman pulling a horse*), participants heard the theme argument mentioned in the auditory prompt (e.g., *what is happening with the horse?*) to prime for passive sentences, whereas the agent argument was mentioned in the auditory prompt (*what is happening with the woman?*) to prime for active sentences. For dative targets (*a boy giving a flower to a teacher*), the agent and the theme were mentioned (e.g., *what is happening with the boy and flower?*) when priming for PO dative sentences, and the agent and the goal were mentioned (e.g., *what is happening with the boy and teacher?*) when priming for DO sentences. The agent was provided in both types of prime prompts to keep participants from starting the sentence with the theme (e.g., *the flower was given to the teacher by the boy*) or with the goal (e.g., *the teacher received the flower from the boy*). For intransitive filler items, a neutral probe (*what’s happening in the picture?*) was provided. A set of six practice trials preceded the experimental items. No feedback on response accuracy was provided for experimental items.

For each trial, the auditory prime was played through speakers, and then a fixation cross was presented in the center of the computer screen for 300 ms (Figure 1). The target picture was then presented concurrently with a beep sound 100 ms in duration. Participants were instructed to describe what was happening in the target picture using a single sentence and start talking as quickly as possible. Participants did not receive instructions regarding what structures they should use in their responses. Once the participant finished their response, they pressed the spacebar on the keyboard to advance to the next trial.

Stimuli were presented on a 24-inch monitor using Experimental Builder (SR research), which was also used to record verbal responses. Eye fixations were recorded using an EyeLink 1,000 Plus desktop remote monocular eyetracker (SR Research, Ottawa, ON, Canada) sampling at 500 Hz. Data collection took place in a room with consistent lighting conditions. Participants were seated approximately 24 inches from the display monitor. A nine-point calibration was conducted before the experiment with a maximum measurement error of 1 degree of visual angle for each calibration point. Fixations were computed by the EyeLink standard algorithm, which parses eye movement data into fixations versus saccades and blinks based on the thresholds for velocity (less than 30 degrees per second), acceleration (less than 8,000 degrees per second squared), motion (less than 0.15 degrees), and/or presence of pupil data. For data analyses, only the fixations that happened during target picture description, as defined by the stimulus presentation onset time and the participant’s pressing a keyboard button to advance to the next trial, were extracted. We accepted fixations of all durations within this trial time window. Participants’ eye fixations were later aligned with their speech data to track changes in their gaze position during sentence production (see section 2.4.2. for further details).

### 2.4 Data analysis

#### 2.4.1 Sentence production data

Sentence production accuracy was computed for each participant. For transitive target pictures, a response was scored as correct if it was an active or passive sentence. For dative pictures, either a prepositional object (PO) or double object (DO) dative response was scored as correct. Variations in verb forms (e.g., *kicks/kicked/is kicking/was kicking*) were accepted, except for passive responses in which the production of an auxiliary verb and a past participle of the main verb was required, followed by a by-phrase (e.g., *is/was kicked by*). Truncated passives were not accepted as correct responses, given that this study measured eye fixations to each character in the scene. For dative targets, responses with pronominal recipients (e.g., *the man is giving her a gift*) were accepted, except for responses where the correct thematic role assignment could not be determined (e.g., *he is giving the ball to him*). Responses containing other, non-target structures (e.g., *the girl saw the jack-in-a-box come out* for *the jack-in-the-box scared the girl*) were scored as incorrect. Each participant’s data were coded by two trained coders, with disagreements resolved by consensus with the second author of this paper.

Only correct responses were included in the data analysis. Each correct response was binarized according to the sentence type produced: 1 (active, PO) or 0 (passive, DO). Transitive and dative responses were then analyzed separately. Given that only two alternating syntactic choices were possible for correct responses, a significant lexical priming effect would be reflected in the increased probability of passive sentence production (out of both active and passive responses) in the theme prime condition compared to the agent prime condition for transitive trials. Similarly, for dative trials, the increased probability of DO sentence production (out of both PO and DO responses) in the goal prime condition compared to the theme prime condition would reflect a significant lexical priming effect.

#### 2.4.2 Eye movement data

Before analysis, fixations were tallied into fixations to the agent and theme characters for transitive trials or fixations to the theme and goal characters for dative trials. These areas of interest (AOI) were pre-defined during the experimental set-up using free-shaped line drawings surrounding each character with approximately 2 visual degrees of margin. Individual fixations that fell within an AOI before the participant’s eye moved out of the AOI were summed to compute the total fixation duration for all AOIs in each trial. Fixations that fell outside of the AOIs were excluded from the analyses.

To align eye fixation data with verbal responses, temporal boundaries for each word were marked in Praat (Boersma and Weenik, 2024) using acoustic and spectrogram analyses. A group of trained coders (2 senior and 2 junior coders) marked these temporal boundaries. The senior coders had demonstrated inter-rater agreement rates of 96 and 97% for all onset times of beep and all target nouns produced in each sentence on a training dataset consisting of responses from three participants that were not included in the analysis (*r*’s > 0.96, *p* < 0.0001). A disagreement was defined by a difference greater than 50 milliseconds between the two coders’ measures. The coders worked on each participant’s data in pairs (1 junior coder, 1 senior coder), with disagreements resolved by consensus.

Eye fixations were then binned based on sentence regions: for transitive responses, sentence regions were defined as 0 to 400 ms (where zero represents the onset of the beep sound and the picture stimulus, which were simultaneous), 400 to 800 ms after picture onset, 800 ms to the onset of the subject noun (hereafter called N1), N1 to the onset of the object noun (hereafter called N2), and N2 to the end of the trial. For dative responses, sentence regions included the same as those for transitive responses, with the addition of N2 to the third sentence argument (hereafter called N3). Sentence regions were defined based on the eye movement patterns reported in the previous literature (Gleitman et al., 2007; Griffin and Bock, 2000; van de Velde et al., 2014).

For the analysis using generalized additive mixed models (GAMMs), a time series dataset was generated by aggregating eye fixations into 25 ms bins. Each fixation was coded either as ‘1’ (fixation to the target AOI) or ‘0’ (fixation to the non-target AOI). This created a dataset wherein each 25 ms bin contained fixations scored as either ‘0’ or ‘1’ for each sentence region. These binary data were then entered into statistical analysis. A significant priming effect was defined as a significant increase in the probability of fixations to the theme AOI over time in the theme prime condition compared to the agent prime condition for transitive trials and an increased probability of fixations to the goal AOI in the goal prime compared to the theme prime condition for dative trials.

In addition, for the analysis of the effects of working memory scores on eye fixations, the proportion of fixation time to AOI was calculated for each region in each trial. For transitive trials, the proportion of fixations to the theme out of total fixations to agent and theme AOIs was calculated. For dative trials, the proportion of fixations to the goal AOI out of total fixations to the theme and goal AOIs was calculated. Given that the fixation time calculations considered only two AOIs, an increase in the proportion of fixation time to one AOI necessarily indicated a proportional decrease in the fixation time to the other AOI within the speech region. Therefore, using fixations to an arbitrarily selected AOI (theme for transitives; goal for datives) as a reference level, we binarized the proportion data for each trial by speech region (Lee, 2020; Mack et al., 2017; Mack and Thompson, 2017). For example, sentence regions with proportion fixation times to theme that were greater than 0.50 were coded as ‘1’, while those with proportions less than 0.50 were coded as ‘0’. These binarized data were entered into statistical analyses.

#### 2.4.3 Statistical analysis

For offline sentence production data, responses were analyzed using mixed effects binary logistic regression models in R (R Core Team, 2021; Posit Team, 2024). Models were fitted using the lme4 package (Bates et al., 2015), and *p* values were generated using the *lmerTest* package (Kuznetsova et al., 2017). Pairwise comparisons (Tukey) with Bonferroni correction were computed using the *emmeans* package (Lenth, 2023). All models included the fixed factors group, prime type, and their interaction. During model fitting, several iterations of each model were fitted using different combinations of random intercepts and slopes by participants and items conditioned on prime. For each model, conditional *R*^2^ (*R*^2^*_c_*) and marginal *R*^2^ (*R*^2^*_m_*) were calculated to measure the extent to which each model captures the variability in the data. Models containing random intercepts and slopes for participants and items outperformed models containing random slopes and intercepts either for participants or items individually. Therefore, all statistical models used to analyze the offline sentence production data included random intercepts and slopes for participants and items conditioned on prime.

Eye fixation data were analyzed using generalized additive mixed modeling following the method employed by Wieling (2018). Generalized additive mixed models (GAMMs) were chosen because they model non-linear effects of one or more independent variables on a dependent variable, such as fixations to a target picture. In this case, GAMMs were fitted to identify non-linear effects of prime condition and age group on fixations to the target AOI across the time course of a sentence production trial. Models were constructed using the mgcv package in R (Wood, 2017; Wieling, 2018) and fitted using the fREML (fast restricted maximum likelihood estimation) method. Since the data were time series data, all models included an autocorrelation of residuals error model [calculated using the acf_resid() function in the mgcv package] to account for the correlation between eye fixations at subsequent time points. Follow-up comparisons of condition means were calculated using the compare_avg() function from the marginaleffects package (Arel-Bundock et al., Forthcoming). During model fitting, the random effects structure was determined by comparing models with and without by-participant and by-item random effects based on smoothing parameter fREML scores that were calculated using compareML() from the itsadug package (van Rij et al., 2022). In all cases, models that included by-participant and by-item random intercepts and slopes outperformed models that did not, according to smoothing parameter fREML score differences between the models. Therefore, all models included by-participant and by-item random intercepts and slopes.

For the transitive and dative datasets, the GAMM analysis unfolded as follows: First, we ran confirmatory models to test whether the data were indeed non-linear over the course of the trial. An independent variable with four levels (called ‘PrimeGroup’) was created representing the four experimental conditions of interest. For example, in the transitive dataset, the ‘OA-theme’ level of PrimeGroup represented fixations to the theme character by older adults in the theme-prime condition. The remaining levels of PrimeGroup represented the three remaining logically possible combinations of group and prime condition (i.e., OA-agent, YA-theme, and YA-agent). A GAMM with fixations to the target AOI as the dependent variable was fitted containing a thin plate regression spline over time for each level of PrimeGroup. Output from this model confirmed non-linearity of our dependent variable in each PrimeGroup condition. The same procedure was carried out for the dative dataset using the ‘theme’ and ‘goal’ prime conditions.

Next, for our primary analyses, we fitted additional models for each sentence region. First, we fitted a model containing binary difference smooths to test the contrasts between the two prime conditions within each group. The significance of these smooth terms indicated the presence or absence of significant priming effects within the groups. Next, we compared the priming effect observed in the older adult group to the priming effect observed in the younger adult group to determine whether the two groups’ priming effects were similar in magnitude and duration (for an analysis using similar reasoning, see Wieling, 2018). We also conducted follow-up pair-wise comparisons of the group means within each prime condition to see whether group differences in fixations were more prevalent in one of the prime conditions.

Lastly, to test our exploratory question of whether participants verbal working memory may interact with lexical priming effects on off-line sentence production and early eye fixation data, a set of mixed effects binary regression models was conducted within each group. The models included forward and backward total scores from the picture-pointing span test, prime condition, and their interactions as fixed factors. All models included random intercepts for participants and items and random slopes for participants and items conditioned on prime.

## 3 Results

Overall, both older and younger adults showed relatively high production of target sentences, with no significant group differences. For transitive pictures, the older adults and younger adults produced 79 and 83% correct target sentences, respectively (*β* = 0.26, *SE* = 0.19, *z* = 1.35, *p* = 0.18). For dative pictures, both groups produced correct target sentences in 90% of trials (*β* = 0.04, *SE* = 0.29, *z* = 0.14, *p* = 0.90). Error types mostly consisted of the production of non-target structures (e.g., ‘the horse is not wanting to move’ for ‘the horse is being pulled by the woman’).

### 3.1 Priming effects on offline sentence production

#### 3.1.1 Transitive sentences

**Figure 2:**
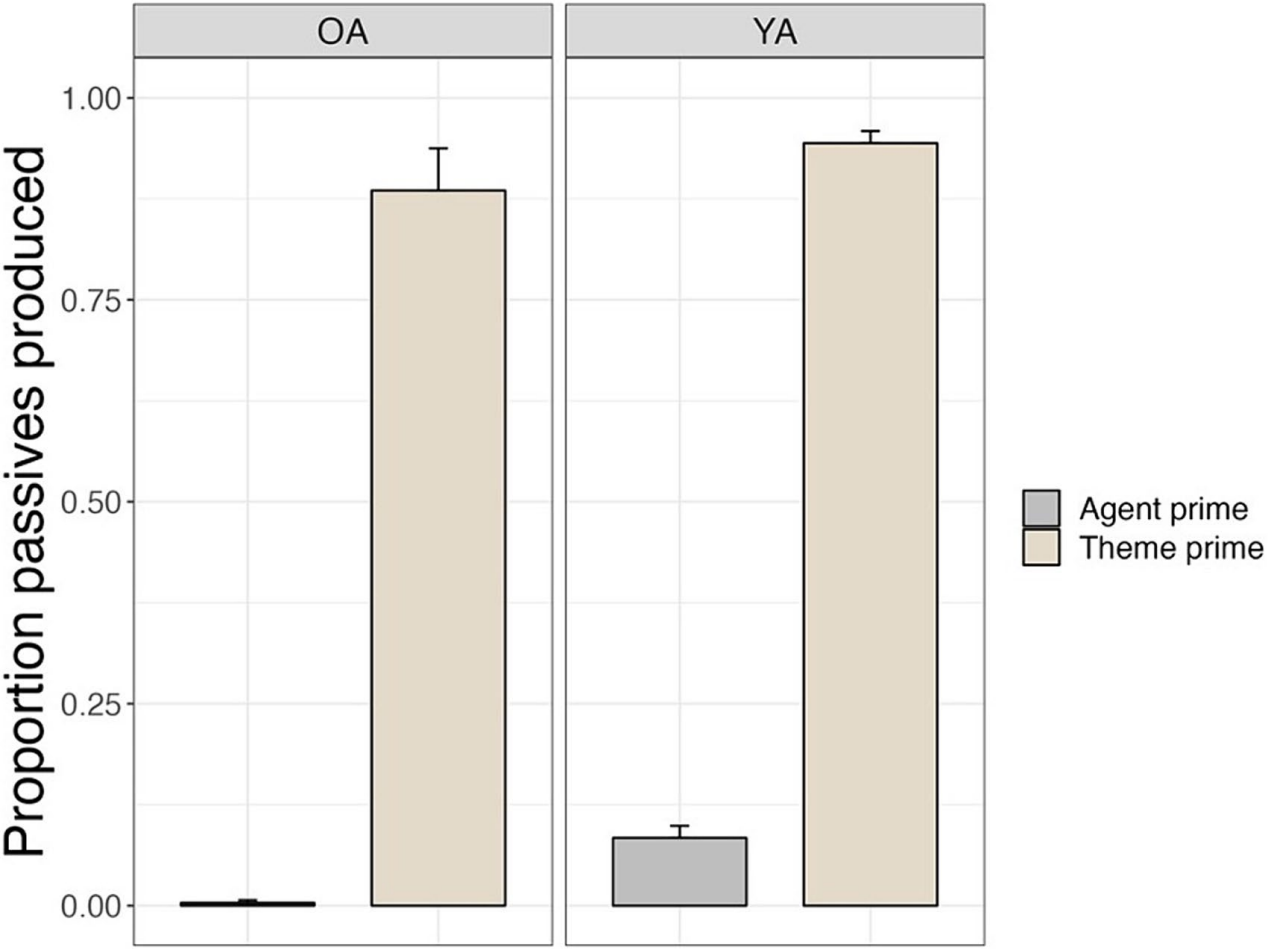
Priming effects on offline sentence production for transitive targets.

Figure 2 shows the proportion of passive productions out of total correct transitive (active and passive) productions for older adults and younger adults under different lexical prime conditions. Both groups displayed significant priming effects, producing more passive sentences in the theme prime condition (Older adults: 89%, *SE* = 5%; Younger adults: 94%, *SE* = 1.5%) compared to the agent prime condition (Older adults: 1%, *SE* = 1%; Younger adults: 8%, *SE* = 1.5%; *β* = 9.13, *SE* = 0.82, *z* = 11.09, *p* < 0.0001). Younger adults produced passive sentences more frequently compared to older adults overall (*β* = 2.76, *SE* = 0.74, *z* = 3.73, *p* = 0.0002), and the interaction between group and prime type was also significant (*β* = −2.20, *SE* = 1.02, *z* = 2.15, *p* = 0.03). However, within-group *post hoc* tests indicated that both groups showed significant priming effects (older adults: *β* = −10.12, *SE* = 1.31, *z* = 7.70, *p* < 0.0001; younger adults: *β* = −7.92, 1.10, z = 7.23, *p* < 0.0001). Group contrasts by priming condition revealed that the prime-by-group interaction was caused by the relatively small number of passives produced by older adults in the agent prime condition (*β* = −2.762, *SE* = 0.690, *z* = −4.005, *p* = 0.0004), while the two groups did not differ in their frequency of passive production in the theme prime condition (*β* = −0.561, *SE* = 0.691, *z* = −0.811, *p* = 0.8492).

#### 3.1.2 Dative sentences

**Figure 3:**
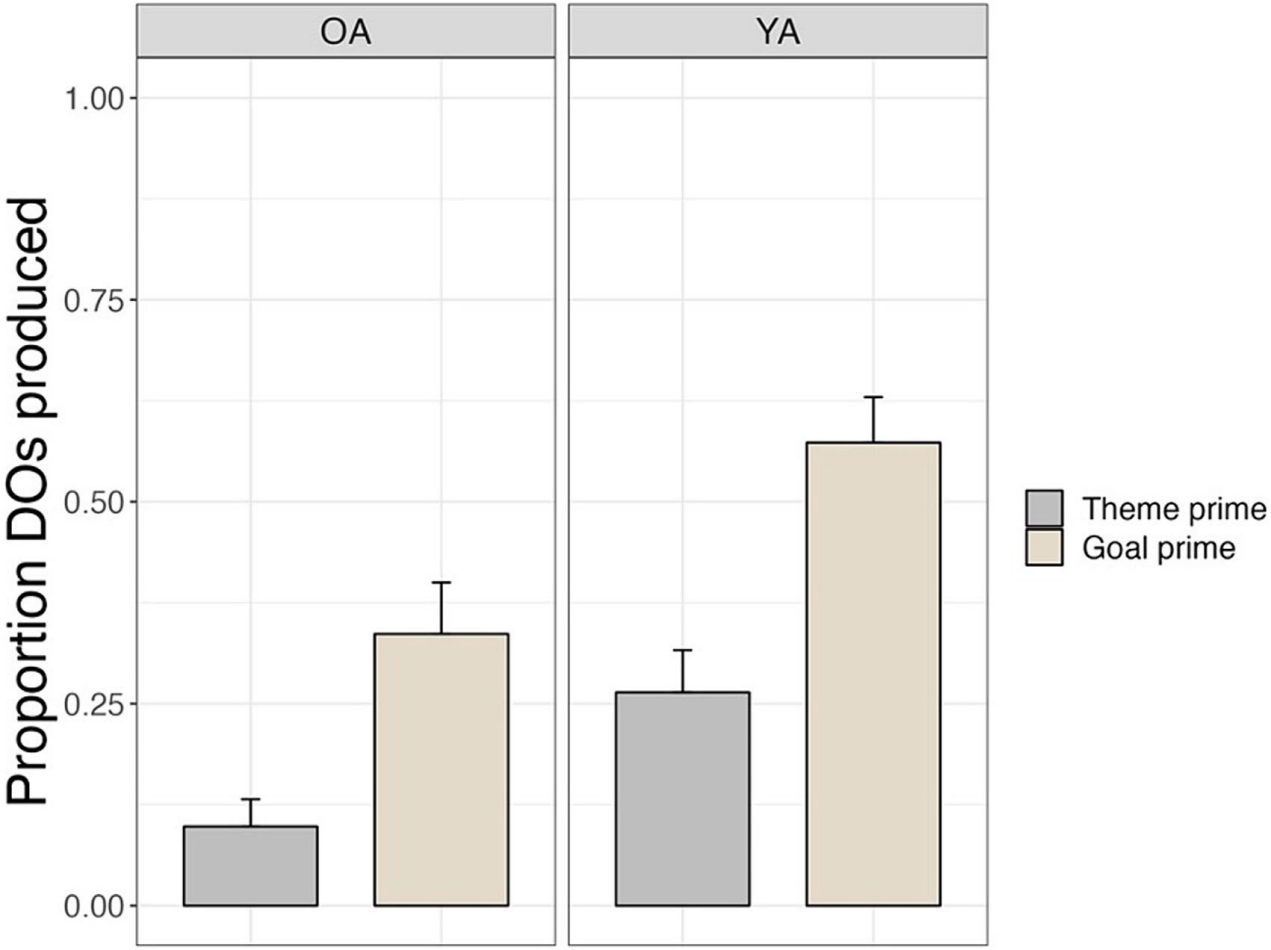
Priming effects on offline sentence production for dative targets.

Figure 3 shows the proportion of double object (DO) productions out of total dative (double object and prepositional object) productions for older and younger adults in the goal prime condition vs. the theme prime condition. Both groups displayed priming effects, producing significantly more DO sentences in the goal prime condition (Older adults: 34%, *SE* = 6%; Younger adults: 57%, *SE* = 6%) compared to the theme prime condition (Older adults: 10%, *SE* = 3%; Younger adults: 26%, *SE* = 5%; *β* = 2.33, *SE* = 0.48, *z* = 4.81, *p* < 0.0001). Younger adults produced more DO sentences overall compared to older adults (*β* = 1.94, *SE* = 0.66, *z* = 2.92, *p* = 0.004). The interaction between group and prime condition was not significant (*β* = −0.39, *SE* = 0.48, *z* = −0.81, *p* = 0.42). *Post hoc* tests showed that older and younger adults both showed significant priming effects (older adults: *β* = −2.34, *SE* = 0.48, *z* = 4.88, *p* < 0.0001; younger adults: *β* = −1.96, *SE* = 0.38, *z* = 5.20, *p* < 0.0001).

### 3.2 Priming effects on eye fixations

#### 3.2.1 Transitive sentences

**Figure 4:**
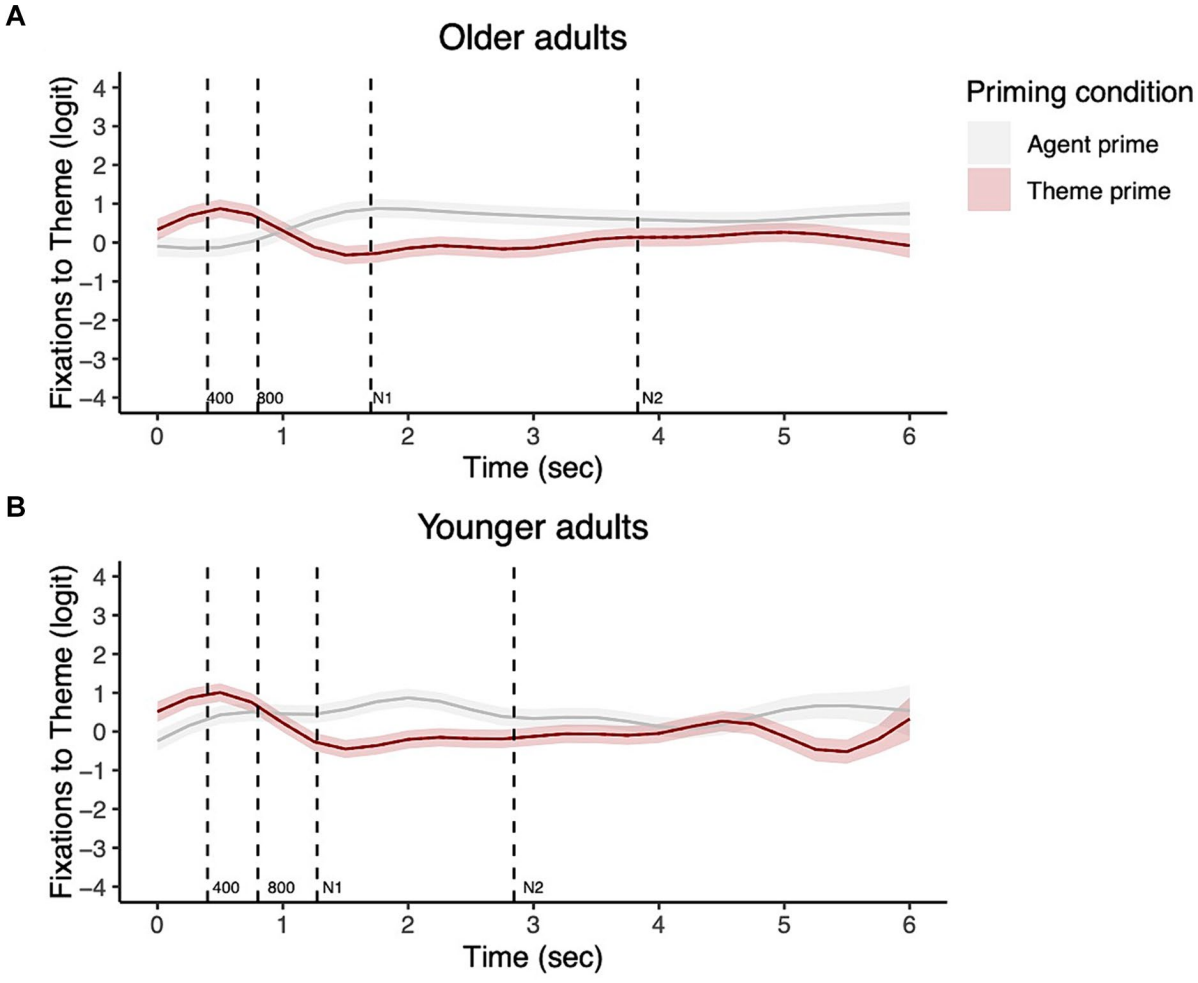
Eye fixations to the theme during the entire sentence trial from picture onset for transitive targets in older adults **(A)** and younger adults **(B)**.

Figure 4 shows the changes in fixations to the theme character over the course of the trial (from picture onset) by prime condition for each group. The output confirmed that there were significant non-linear changes in fixations to the theme in all four prime conditions (Agent prime condition-Older adults: EDF = 8.65, Ref. DF = 8.97, *χ*^2^ = 309.76, *p* < 0.001; Theme prime condition-Older adults: EDF = 8.49, Ref. DF = 8.93, *χ*^2^ = 269.15, *p* < 0.001; Agent prime condition-Younger adults: EDF = 7.94, Ref. DF = 8.64, *χ*^2^ = 162.23, *p* < 0.001; Theme prime condition-Younger adults: EDF = 8.82, Ref. DF = 8.99, *χ*^2^ = 183.95, *p* < 0.001).

**Figure 5:**
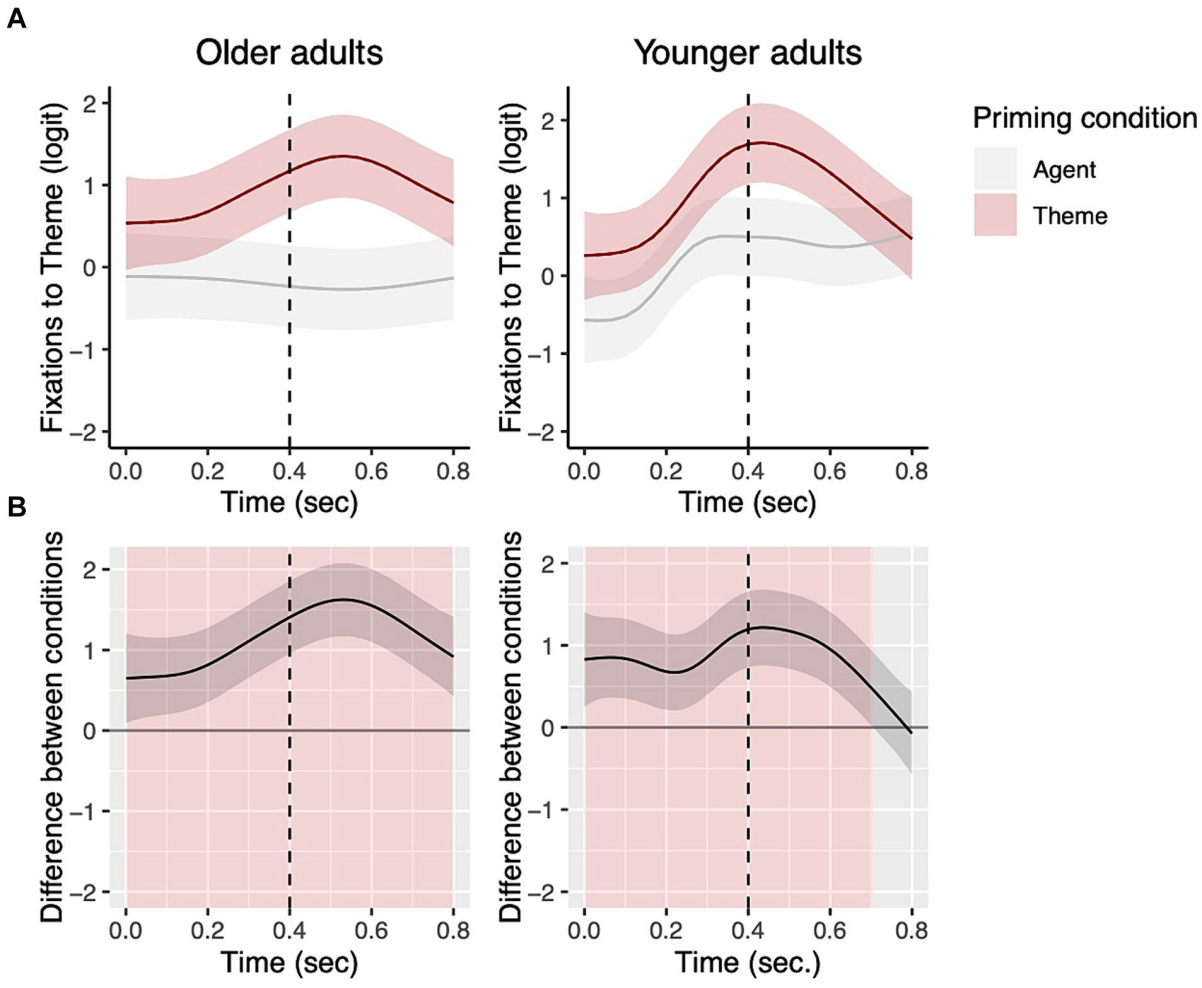
Priming effects on eye fixations from picture onset to 800 ms for transitive targets. The top panels show fixations to the theme by prime condition for each group with the dotted vertical line indicating 400 ms **(A)**. Shading in the bottom panels **(B)** represent areas where the difference smooth confidence interval does not include zero.

In order to measure whether the participants showed differences in their fixations to the theme from the earliest time window, we next focused on the first two sentence regions (i.e., picture onset −400 ms and 400–800 ms.). The top panels of Figure 5 show fixations to the theme by prime condition from picture onset to 800 ms. The bottom panels (Figure 5B) additionally show the difference in fixations to the theme between priming conditions for each group. Areas highlighted in red are periods where the fixations to the theme differed significantly between the two priming conditions.

For each time window, the models first tested whether each group showed significant priming effects. There was a significant priming effect for both groups in the 0–400 ms time window such that the participants showed more fixations to the theme in the theme vs. agent prime condition, as shown in Figure 5A (Older adults: EDF = 2.00, Ref. DF = 2.00, *χ*^2^ = 7.43, *p* = 0.02; Younger adults: EDF = 4.10, Ref. DF = 5.01, *χ*^2^ = 23.68, *p* = 0.0003). In the 400–800 ms time window, both groups showed significant priming effects (Older adults: EDF = 5.90, Ref. DF = 7.23, *χ*^2^ = 51.20, *p* < 0.001; Younger adults: 7.42, Ref. DF = 8.76, *χ*^2^ = 156.64, *p* < 0.001). The statistical results for the remaining time windows (800-N1, N1–N2, N2-end of trial) are reported in Supplementary Table S1. There were still significant differences in fixations to the theme between the prime conditions, but in the direction that participants showed greater fixations to the theme in the agent vs. theme condition, as shown in Figure 4.

When comparing priming effects between the two groups in the 0–400 ms time window, the contrast between the two groups’ priming effects was not significant, indicating that the two groups showed priming effects of similar duration in this time window (EDF = 2.85, Ref. DF = 3.36, *χ*^2^ = 2.10, *p* = 0.62, Figure 5B). In the 400–800 ms window, however, the contrast between the two groups’ priming effects was significant (EDF = 4.89, Ref. DF = 5.50, *χ*^2^ = 42.73, *p* = <0.001, Figure 5B). This group difference was because while older adults continued to show the significant priming effect for the entirety of the 400–800 time window, young adults stopped showing a significant difference in their fixations between the priming conditions earlier, before 800 ms, as shown in Figure 5B.

**Figure 6:**
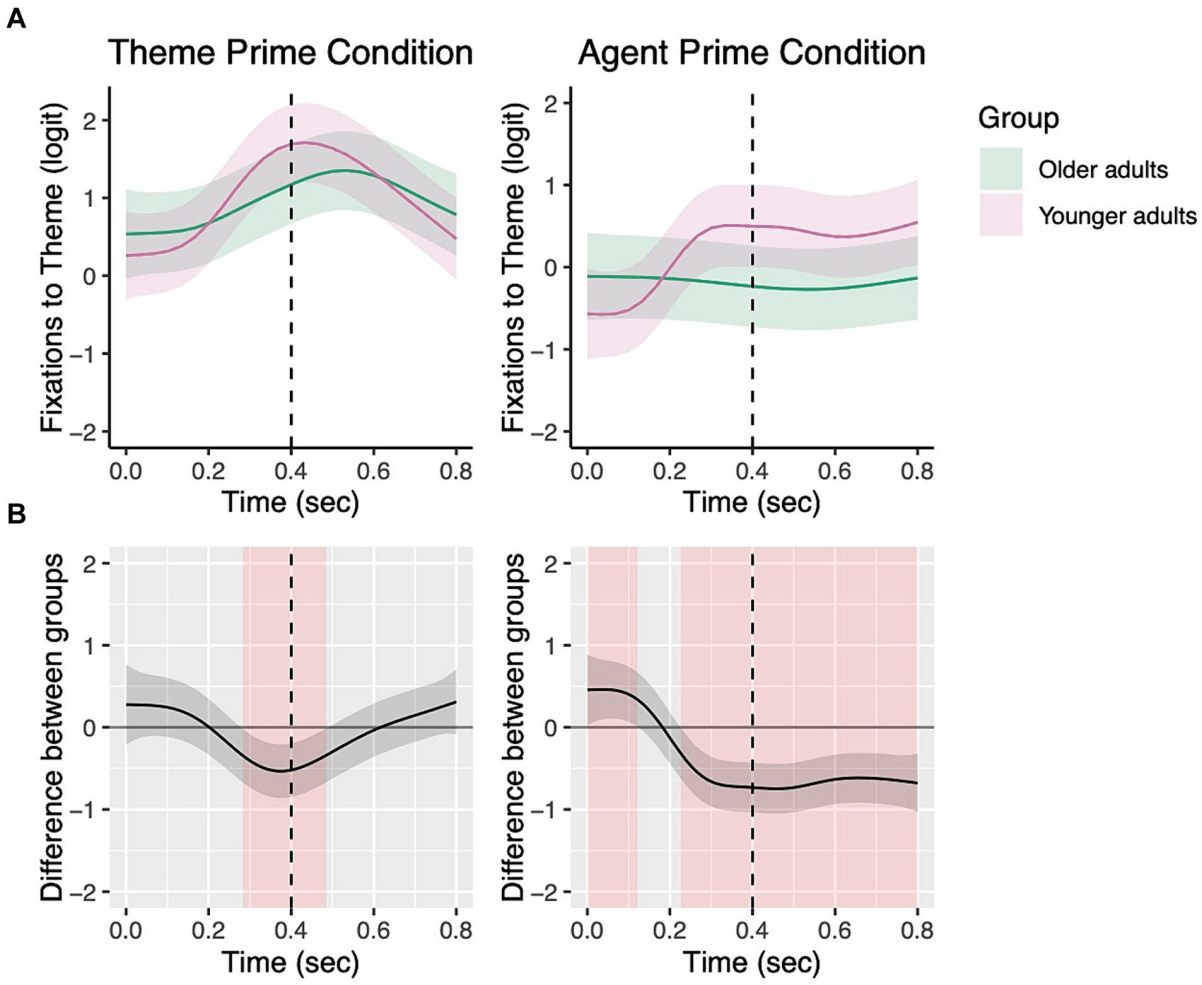
Group comparisons in the theme and agent prime conditions from picture onset to 800 ms for transitive targets. The top panels show fixations to the theme by group in each priming condition with the dotted vertical line indicating 400 ms **(A)**. Shading in the bottom panels **(B)** represents areas where the difference smooth confidence interval does not include zero.

Figure 6 presents data from Figure 5 in terms of group comparisons in fixations to the theme character by prime condition. In the 0–400 ms time window, older adults looked to the theme character significantly less than younger adults in the theme prime condition (*β* = 1.12, *SE* = 0.05, *z* = 2.48, *p* = 0.01), and did so marginally less in the agent prime (*β* = 0.09, *SE* = 0.05, *z* = 1.91, *p* = 0.06). In the 400–800 ms time window, the two groups did not differ significantly in their looks to the theme character in the theme prime condition (*β* = 0.05, *SE* = 0.05, *z* = 0.89, *p* = 0.38), but older adults looked at the theme significantly less in the agent prime condition compared to younger adults (*β* = 0.17, *SE* = 0.07, *z* = 2.40, *p* = 0.02).

#### 3.2.2 Dative sentences

**Figure 7:**
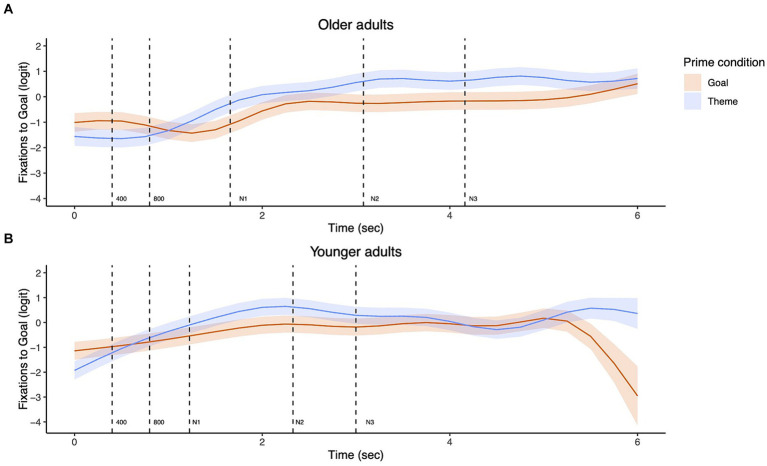
Eye fixations to the goal during the entire sentence trial from picture onset for dative targets in older adults **(A)** and younger adults **(B)**.

Figure 7 shows the non-linear change in fixations to the goal character over the course of a trial. Statistical results confirmed that both younger and older adults showed non-linear changes in their fixations in all conditions (Older adults – Theme prime: EDF = 8.72, Ref. DF = 8.98, *χ*^2^ = 842.20, *p* < 0.001; Older adults – Goal prime: EDF = 8.42, Ref. DF = 8.91, *χ*^2^ = 126.70, *p* < 0.001; Younger adults – Theme prime: EDF = 8.28, Ref. DF = 8.81, *χ*^2^ = 714.50, *p* < 0.001; Younger adults – Goal prime: EDF = 8.80, Ref. DF = 8.98, *χ*^2^ = 275.30, *p* < 0.001).

**Figure 8:**
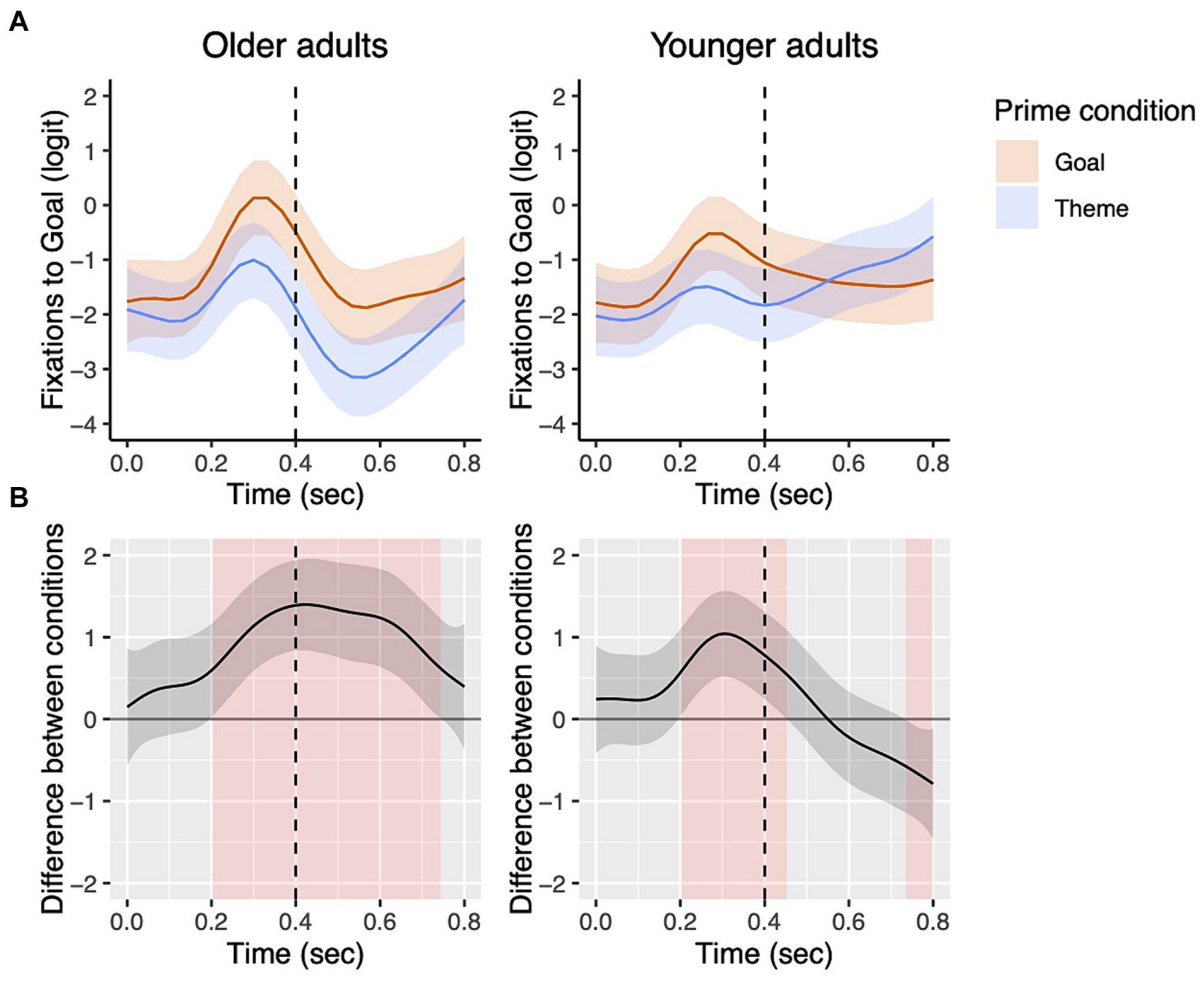
Priming effects on eye fixations from picture onset to 800 ms for dative targets. The top panels show fixations to the goal by prime condition for each group with the dotted vertical line indicating 400 ms **(A)**. Shading represents areas where the difference smooth confidence interval does not include zero **(B)**.

The top panels of Figure 8A show the fixations to the goal character in the goal vs. theme prime condition for the early time windows of our interest. The bottom panels (Figure 8B) additionally show the difference in fixations to the goal between prime conditions for each group. Areas highlighted in red are periods where the fixations to the goal differed significantly between the two prime conditions. As in the transitive data, for each time window, we first examined the priming effect in the older adults and younger adults separately. We then compared the two groups’ priming effects, specifically their difference in duration.

As shown in Figure 8A, in the 0–400 ms time window, both older adults and young adults showed significantly increased fixations to the goal character in the goal prime vs. theme prime condition, i.e., priming effects (Older adults: EDF = 3.98, Ref. DF = 4.87, *χ*^2^ = 45.28, *p* < 0.001; Younger adults: EDF =6.50, Ref. DF = 8.16, *χ*^2^ = 32.92, *p* < 0.001). In the 400–800 ms window, older adults continue to show increased fixations to the goal character in the goal prime condition compared to the theme prime condition (Older adults: EDF = 3.52, Ref. DF = 4.21, *χ*^2^ = 34.29, *p* < 0.001). The effect of prime condition was significant for young adults as well in the 400–800 ms window, but in the opposite direction: they showed decreased fixations to the goal character in the goal prime vs. theme prime condition (Younger adults: EDF = 2.57, Ref. DF = 2.95, *χ*^2^ = 26.81, *p* < 0.001).

The models contrasting the two groups’ priming effects further revealed no significant difference between older and younger adults in the 0–400 ms window (EDF = 3.55, Ref. DF = 4.24, *χ*^2^ = 4.26, *p* = 0.41). However, in the 400–800 ms time window, there was a significant difference between the two groups’ priming effects (EDF = 3.54, Ref. DF = 4.24, *χ*^2^ = 15.20, *p* = 0.006). As shown in Figure 8B, the priming effect for older adults was longer in duration compared to younger adults. Additionally, younger adults showed a difference between conditions (695–776 ms), but in the opposite direction. In other words, older adults continued to show a significant priming effect, while the priming effect for younger adults ceased to be significant.

**Figure 9:**
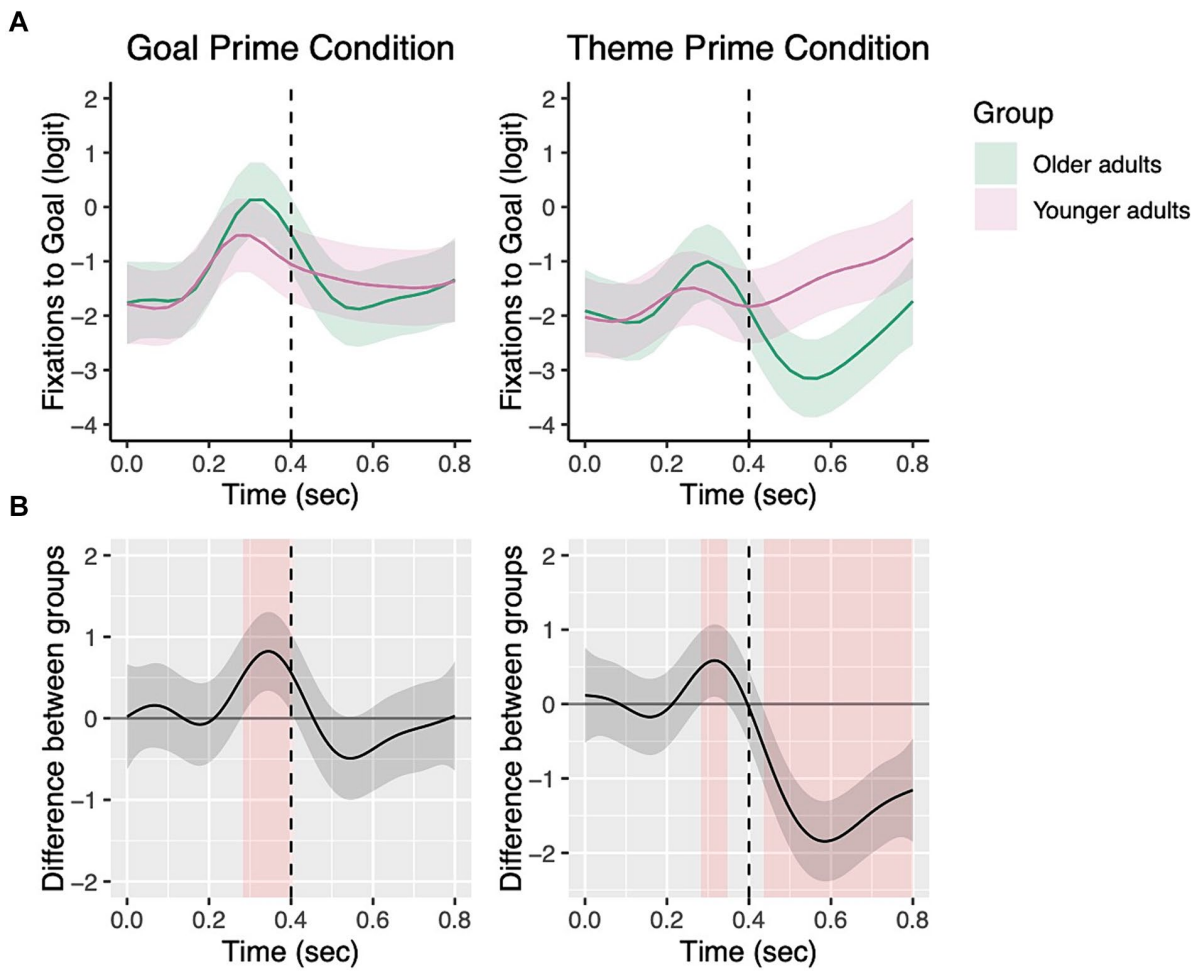
Group comparisons in the goal and theme prime conditions from picture onset to 800 ms for dative targets. The top panels show fixations to the goal by group in each priming condition with the dotted vertical line indicating 400 ms **(A)**. Shading in the bottom panels **(B)** represents areas where the difference smooth confidence interval does not include zero.

Figure 9 presents data from Figure 8 in terms of group comparisons in fixations to the goal character by prime condition. In the 0–400 ms time window, the two groups did not show differences in fixations to the goal in the goal prime condition (*β* = −0.05, *SE* = 0.04, *z* = 1.47, *p* = 0.14) or in the theme prime condition (*β* = −0.04, *SE* = 0.03, *z* = −1.23, *p* = 0.22). In the 400–800 ms time window, there was no group difference in the goal prime condition (*β* = −0.03, *SE* = 0.05, *z* = −0.60, *p* = 0.55), but in the theme prime condition, older adults fixated the goal less than younger adults (*β* = 0.16, *SE* = 0.05, *z* = 3.26, *p* = 0.001).

### 3.3 Verbal working memory and priming effects

Exploratory analyses tested whether individual participants’ verbal working memory scores interacted with the priming effects. For offline sentence production data, there was no main effect of working memory and no significant interactions between working memory scores (either forward or backward) and prime conditions (all *p*’s > 0.15). This was true in both groups for both transitive and dative sentences. The full results are reported in Supplementary Table S3.

The statistical results focusing on the interactions between working memory and priming effects on eye fixations are reported in Table 3. For the transitive sentences, priming effects did not significantly change as an effect of working memory scores in the 0–400 ms region for either group. Similarly, there were no interactions between working memory scores and prime for either group in the 400–800 ms region. Turing to the eye fixations for dative sentences, in the 0–400 ms region, both working memory forward and backwards scores significantly interacted with the prime effects in older adults, such that older adults with lower working memory scores showed smaller priming effects in their eye fixations compared to the older adults with higher working memory scores. In the 400–800 ms region, older adults with lower working memory backward scores continued to gaze at the primed character, thus resulting in longer priming effects, whereas those with higher working memory scores ceased to fixate the primed character. For younger adults, working memory scores did not show any significant interaction with priming effects across all regions. In addition, although not a main analysis of our interest, no main effects of working memory were significant in either group, except for a main effect of forward scores in young adults’ 0–400 ms region (*p* = 0.02) for dative targets. For full output (see Supplementary Table S4).

**Table 3:** Interactions between verbal working memory scores and priming effects in eye fixations in the 0–400 ms and 400–800 ms time window.

	Transitive sentences							
	0–400 ms				400–800 ms			
	*β*	*SE*	*z*	*p*	*β*	*SE*	*z*	*p*
Older adults								
WMF × Prime	0.07	0.21	0.38	0.71	0.06	0.21	0.29	0.77
WMB × Prime	0.02	0.21	0.10	0.91	−0.14	0.21	−0.69	0.49
Younger adults								
WMF × Prime	0.26	0.21	1.21	0.23	−0.11	0.21	−0.51	0.61
WMB × Prime	0.36	0.20	1.78	0.08	−0.38	0.21	−1.82	0.07

Dative sentences								
	0–400 ms				400–800 ms			
	*β*	*SE*	*z*	*p*	*β*	*SE*	*z*	*p*
Older adults								
WMF × Prime	0.87	0.28	3.10	0.002**	−0.21	0.31	−0.69	0.49
WMB × Prime	0.76	0.27	2.77	0.006**	−0.72	0.32	−2.26	0.02*
Younger adults								
WMF ×Prime	−0.48	0.24	−1.98	0.05	−0.39	0.27	−1.42	0.16
WMB × Prime	−0.15	0.26	−0.59	0.56	−0.30	0.26	−1.14	0.25

WMF, working memory forward score; WMB, working memory backward score. ** p <.01; * *p* <.05.

## 4 Discussion

The purpose of the study was to examine if and how aging impacts the processes of lexical encoding and syntactic formulation involved in sentence production. Specifically, we measured the effects of auditory lexical priming on the syntactic choices that older and younger speakers make during the production of transitive (active/passive) and dative (double object/prepositional object) sentences and on-line eye fixations to primed characters during early sentence planning. In addition, we examined if individual participants’ verbal working memory scores, as obtained by a picture pointing span task (DeDe et al., 2014), would interact with speakers’ syntactic and lexical processing abilities.

The results from the offline sentence production data revealed no effect of aging, indicating robust syntactic flexibility in older adults. Older adults showed comparable lexical priming effects as younger adults on the choices of syntactic structures that they produced, even though the older adult group produced fewer passives and fewer double object (DO) dative sentences across the board compared to younger adults. Less frequent production of those non-canonical sentences in older adults is consistent with the widespread finding that older adults tend to produce fewer complex sentences compared to younger adults (e.g., Agmon et al., 2022; Kemper, 1987; Kemper et al., 2001, 2003). Nonetheless, older adults produced the structure that allows earlier mention of the primed word to the same degree as younger adults, producing more passives when they heard the theme noun in the auditory prime probe compared to when they heard the agent noun word. Similarly, for dative target pictures, older adults produced the DO structure more frequently when primed with the goal noun compared to the theme noun.

However, age-related differences were significant when lexical priming effects were measured in participants’ eye fixations. Older adults began to show priming effects on eye fixations as early as young adults, showing significantly greater looks to the character that was primed. However, their duration of the priming effects was significantly longer than that of young adults in both transitive and dative sentences. In transitive sentences, both groups showed significantly greater looks to the theme during 0–400 ms after hearing the theme prime than the agent prime (Figure 5), and the group difference in fixations to the theme was barely different between the groups. However, during the 400–800 ms time window, while older adults continued to show greater looks to the theme in the theme prime condition, younger adults’ priming effects ceased to be significant at around 700 ms, indicating earlier completion of lexical encoding (Figure 5). Between-group comparisons further elucidated that the longer priming effects found in older adults’ fixations are not because older adults failed to look at the theme in the theme prime condition, but they looked at the theme less than young adults in the agent prime condition (Figure 6).

For dative sentences, similarly, both groups showed early priming effects from the picture onset (0–400 ms), more specifically starting at around 200 ms after picture onset, as shown in Figure 8. Between-group comparisons showed that both groups looked at the goal significantly more in the goal prime condition during this early 200–400 ms window. In the 400–800 ms time window, older adults continued to show a priming effect, while younger adults ceased to show a priming-induced difference in fixations. Significant group differences in fixations to the goal in the theme prime condition but not in the goal prime condition (Figure 9) further suggest that while older adults generally showed fewer looks to the goal during this 400–800 ms window compared to the 0–400 ms window, they were still looking at the goal AOI substantially more in the goal prime condition compared to the theme prime condition. Taken together, these findings from within and between group comparisons suggest that older adults were as fast as young adults in drawing their visual attention to the primed character; however, they were different in the duration of the priming effects. Such elongated priming effects found in older adults are likely reflective of slower and more effortful encoding of the lexical information, consistent with previous studies showing slower response times on lexical decision tasks or difficulty with lexical retrieval for older adults (Burke et al., 1991; Feyereisen, 1997; James and Burke, 2000; Mortensen et al., 2006; Shafto et al., 2007; Verhaegen and Poncelet, 2013).

Situating the results from our first two questions in the existing literature, the current findings support the claim that aging affects lexical processing more greatly than syntactic processing during sentence production. The significant and robust ability to flexibly produce different syntactic structures in response to varying degrees of lexical accessibility seen in our older adults is in line with previous studies suggesting a preserved ability to access and generate syntactic structures in older adults through sentence construction tasks (Davidson et al., 2003) and structural priming paradigms (Hardy et al., 2017, 2020; Lee et al., 2022). In addition, these findings support the claim that language production operates incrementally so that alternative syntactic configurations can accommodate varying levels of lexical accessibility (Bock and Warren, 1985; Levelt, 1989; Kempen and Hoenkamp, 1987; Slevc, 2011) and suggest that the ability to coordinate and produce words and syntax incrementally remains largely preserved in healthy aging.

Furthermore, our findings suggest that when sentence production abilities in older adults are measured primarily based on offline accuracy and use of frequency measures (e.g., Agmon et al., 2022; Kemper et al., 2001, 2003; Sung et al., 2024), such end-of-the-task measures may not provide sufficient information about how aging affects specific cognitive-linguistic processes of sentence production or how older adults might use different planning strategies in real-time. By observing the dissociated effects of aging on offline production and real-time eye fixation data, the current study could reveal that although older adults did not show difficulty in their overall sentence production measures, real-time lexical processing was more delayed in older adults. Similarly, Hardy et al. (2020), taking advantage of speech initiation time measures, discovered that healthy aging resulted in reduced lexical access and processing in the context of intact syntactic processing. When producing sentences with conjoined noun phrases (e.g., *the spoon and the car move up*), their older adults, similar to their young adults, showed significantly faster speech initiation times when they were primed with a similar syntactic structure (e.g., *the eye and the fish move apart*). However, the older adults showed limited benefit in speech times when the authors manipulated ease of lexical encoding by letting participants preview one (e.g., *spoon*) or both object images (e.g., *spoon* and *car*) before picture description. While younger adults showed preview benefits on their speech time for both images, older adults showed a preview benefit for only one image, indicating their reduced scope of lexical processing.

Lastly, our exploration of the contribution of verbal working memory to lexical and syntactic processes revealed that longer priming effects on eye fixations found in older adults are in part attributable to reduced verbal working memory. Older adults’ working memory scores did not interact with off-line syntactic measures. However, the older adults with lower working memory scores tend to show more prolonged looks to the primed character. But this pattern was significant only for dative targets, not for transitive targets. This finding makes some intuitive sense considering the increased number of noun arguments involved in dative sentences compared to transitive sentences and is in line with previous studies where working memory effects only become significant when task complexity increases (Sung et al., 2017; DeDe et al., 2004). Thus, it may be the case that working memory resources in older adults were sufficiently taxed by increased demands for dative sentences, yielding some reductions in lexical processing. In contrast, young adults’ working memory scores did not influence either offline syntactic production or eye fixation measures. Our experimental tasks, which involved fairly common syntactic alternations, might not have sufficiently taxed the working memory capacity of young adults, as has also been shown in previous studies (e.g., DeDe et al., 2004; Sung et al., 2024; Sung et al., 2017). A more rigorous and systematic investigation of how working memory contributes to the different processes involved in sentence production is warranted. Nonetheless, the current preliminary findings demonstrate that working memory differentially modulates lexical and syntactic processes involved in sentence production and suggest that individual differences in working memory capacity play a more significant role in sentence production as people age.

Different results found between the current study and the other two studies using a similar paradigm (Slevc, 2011; Sung et al., 2024) deserve some attention. Whereas Sung et al. (2024), using an oral reading lexical priming task, found that older adults showed reduced syntactic flexibility, the current study did not show such age effects on off-line syntactic production. This difference could be because the current task presented the lexical prime using a question prompt (‘what’s happening with the horse?’), increasing our older adults’ chances of producing them earlier in the sentence. Unlike Slevc (2011), our young adults did not show working memory effects on their sentence production processes. This discrepancy could be because Slevc (2011) used a concurrent memory load task, where their young adults had to hold two unrelated words in their memory for a delayed recall while describing target pictures. Thus, their task was more taxing than ours, allowing limitations in verbal working memory to exert greater influence on sentence production. Future studies should examine how age-related changes in lexical and syntactic processes involved in sentence production are affected by varying degrees of task and sentence complexity. In addition, the relationships between working memory and subprocesses of sentence production need to be more carefully investigated using experimental conditions under which working memory limitations may exert greater effects on speakers’ performance.

In conclusion, this study examined how healthy aging affects lexical and syntactic processes involved in sentence production. In offline sentence production, older adults flexibly produced the syntactic structure that allows earlier production of more accessible lexical items, indicating that syntactic processes and flexibility are largely preserved in aging. In contrast, older adults showed longer lexical priming effects on early fixations to the primed character, indicating more effortful lexical encoding during sentence planning processes. Such elongated priming effects on eye fixations were more noticeable for older adults with lower working memory scores, especially for dative pictures. However, working memory did not show significant interactions in off-line syntactic production in either group or with eye fixation measures in young adults. Taken together, the current findings indicate that not all processes of sentence production change with healthy aging. Syntactic flexibility for formulating different grammatical structures remains largely robust during aging; however, lexical encoding processes are more susceptible to age-related cognitive changes, with individuals’ verbal working memory playing a greater role in older adults’ lexical processing during sentence production, than in younger adults.

## Data Availability

The datasets presented in this study can be found in online repositories. The names of the repository/repositories and accession number(s) can be found here: https://osf.io/mhr35/?view_only=7b0a10aa49fe45c392e0d362bf6f7d47.
