# Supplementary material for: Syntactic flexibility and lexical encoding in aging sentence production: an eye tracking study
Source: Front Psychol. 2024 Aug 26;15:1304517. doi: 10.3389/fpsyg.2024.1304517 (PMC11381281; doi:10.3389/fpsyg.2024.1304517)
Supplement: Supplementary file 1 [file Table_1.DOCX]

Supplementary Table 1

*Priming effects across different time windows for transitive targets*

| Parametric coefficients: Estimate(Std. Error) | | | |  |  |
| --- | --- | --- | --- | --- | --- |
|  | 0-400ms. | 400-800ms. | 800ms-N1 | N1-N2 | N2-end |
| (Intercept) | −0.23 (0.64) | −0.35 (0.52) | 2.46 (1.34) | 2.30 (0.37)*** | 0.18 (0.27) |
| GroupYA | 0.34 (0.31) | 1.00 (0.41)* | 14.08 (18.54) | −1.52 (0.49)** | −0.37 (0.27) |
| Approx. sig. of smooth terms: EDF(Ref. DF) | | | |  |  |
|  | 0-400ms. | 400-800ms. | 800ms-end | N1-N2 | N2-end |
| EDF: s(Time):GroupOA | 1.00 (1.00) | 3.60 (4.69)* | 8.06 (8.34)*** | 8.19 (8.67)*** | 7.96 (8.67)*** |
| EDF: s(Time):GroupYA | 5.83 (7.20)*** | 1.68 (2.15) | 4.92 (5.12)*** | 8.71 (8.87)*** | 7.52 (8.36)*** |
| EDF: s(Time):OATheme | 2.00 (2.00)* | 5.90 (7.23)*** | 9.29 (9.54)*** | 9.32 (9.76)*** | 8.10 (8.79)*** |
| EDF: s(Time):YATheme | 4.10 (5.07)*** | 7.42 (8.76)*** | 3.37 (3.83) | 9.58 (9.82)*** | 8.85(9.56)*** |
| EDF: s(ID) | 0.00 (38.00)*** | 0.00 (39.00)*** | 13.24 (39.00) | 0.00 (39.00) | 0.01 (37.00) |
| EDF: s(Prime,ID) | 59.72 (78.00)*** | 69.06 (78.00)** | 54.86 (77.00) | 72.05 (78.00)* | 68.19 (78.00)*** |
| EDF: s(Item) | 12.35 (17.00) | 8.32 (18.00) | 0.00 (18.00)*** | 8.85 (17.00) | 0.00 (18.00) |
| EDF : s(Prime,Item) | 19.96(36.00) | 24.00 (36.00) | 30.87 (36.00)*** | 21.27 (36.00) | 29.26 (36.00) |
| AIC | −2234.47 | −4726.22 | −15534.36 | −56409.83 | −21039.12 |
| BIC | −1433.54 | −3756.29 | −14505.44 | −55109.41 | −19909.81 |
| Log Likelihood | 1226.06 | 2488.49 | 7896.02 | 28348.94 | 10654.05 |
| Deviance | 11801.21 | 18025.65 | 28073.51 | 75728.16 | 43379.63 |
| Deviance explained | 0.25 | 0.20 | 0.06 | 0.10 | 0.03 |
| Dispersion | 1.00 | 1.00 | 1.00 | 1.00 | 1.00 |
| R2 | 0.33 | 0.27 | 0.08 | 0.14 | 0.05 |
| GCV score | 9720.60 | 13420.88 | 12387.67 | 28586.64 | 19721.54 |
| Num. obs. | 11607 | 16918 | 122512 | 61617 | 32774 |
| Num. smooth terms | 8 | 8 | 8 | 8 | 8 |
| ∗∗∗p < 0.001; ∗∗p < 0.01; ∗p < 0.05 | | | |  |  |

*Note:* The parameters “OATheme” and “YATheme” correspond to the contrast between priming conditions for older adults and younger adults, respectively

Supplementary Table 2

*Priming effects across different time windows for dative targets*

| Parametric coefficients: Estimate(Std. Error) | | | |  |  |  |
| --- | --- | --- | --- | --- | --- | --- |
|  | 0-400ms. | 400-800ms. | 800ms-N1 | N1-N2 | N2-N3 | N3-end |
| (Intercept) | −1.56 (0.48)** | −2.36 (0.42)*** | −1.77 (5.43) | −0.81 (0.29)** | 1.72 (0.45)*** | 0.37 (0.41) |
| GroupYA | −0.15 (0.35) | 1.27 (0.37)*** | 2.06 (1.12) | 1.73 (0.34)*** | −1.35 (0.36)*** | −0.73 (0.37)* |
| Approx. sig. of smooth terms: EDF(Ref. DF) | | | |  |  |  |
|  | 0-400ms. | 400-800ms. | 800ms-end | N1-N2 | N2-N3 | N3-end |
| EDF: s(Time):GroupOA | 9.09 (11.24)*** | 6.09 (7.39)*** | 7.68 (8.05)*** | 8.24 (8.74)*** | 7.72 (8.39)*** | 7.56 (8.22)*** |
| EDF: s(Time):GroupYA | 10.23 (12.15)*** | 3.77 (4.84)*** | 4.28 (4.85)*** | 7.52 (8.03)*** | 7.15 (8.12)** | 8.71 (8.94)*** |
| EDF: s(Time):OAGoal | 3.98 (4.88)*** | 3.52 (4.21)*** | 7.82 (8.38)*** | 8.95 (9.60)*** | 8.40 (9.18) | 8.73 (9.38)*** |
| EDF: s(Time):YAGoal | 6.50 (8.16)*** | 2.57 (2.94)*** | 5.41 (5.78)*** | 8.58 (8.93)*** | 9.02 (9.15)* | 6.39 (7.52)*** |
| EDF: s(ID) | 23.04 (39.00) | 20.94 (38.00) | 27.42 (39.00)*** | 24.66 (38.00) | 23.81 (37.00) | 16.77 (38.00) |
| EDF: s(Prime,ID) | 40.85 (78.00) | 41.55 (78) | 40.32 (78)* | 43.08 (78) | 45.61 (78.00) | 53.24 (78) |
| EDF: s(Item) | 13.18 (18.00) | 13.24 (18.00) | 0.00 (18.00) | 0.00 (17.00)*** | 6.73 (18.00) | 5.34 (18.00) |
| EDF: s(Prime,Item) | 17.20 (36.00) | 14.56 (36.00) | 33.98 (36.00)*** | 30.76 (36.00)* | 25.30 (36.00) | 26.04 (36.00) |
| AIC | −8294.37 | −3167.61 | −12673.38 | −30110.87 | −26371.39 | −16691.38 |
| BIC | −7334.78 | −2364.78 | −11666.63 | −28901.93 | −25234.69 | −15600.79 |
| Log Likelihood | 4276.10 | 1695.66 | 6467.62 | 15192.75 | 13323.81 | 8481.84 |
| Deviance | 9860.20 | 7941.66 | 17853.92 | 66989.80 | 30921.66 | 26442.02 |
| Deviance explained | 0.32 | 0.25 | 0.07 | 0.02 | 0.15 | 0.14 |
| Dispersion | 1.00 | 1.00 | 1.00 | 1.00 | 1.00 | 1.00 |
| R2 | 0.36 | 0.26 | 0.13 | 0.04 | 0.18 | 0.18 |
| GCV score | 7547.88 | 7376.05 | 8703.66 | 30526.48 | 12412.45 | 12213.00 |
| Num. obs. | 12629 | 9675 | 16147 | 49246 | 27724 | 22247 |
| Num. smooth terms | 8 | 8 | 8 | 8 | 8 | 8 |
| ∗∗∗p < 0.001; ∗∗p < 0.01; ∗p < 0.05 | | | |  |  |  |

*Note:* The parameters “OAGoal” and “YAGoal” correspond to the contrast between priming conditions for older adults and younger adults, respectively

Supplementary Table 3

*The interaction of working memory scores with prime effects in offline sentence production (WMF = Working memory forward score; WMB = Working memory backward score)*

| Transitive sentences | | | | | | | | | | | |
| --- | --- | --- | --- | --- | --- | --- | --- | --- | --- | --- | --- |
|  | **β** | | | **SE** | ***z*** | | | | | ***p*** | ***R^2^*** |
| Older adults |  | |  | | |  | | |  | |  |
| WMF | -0.28 | | 0.67 | | | -0.42 | | | 0.68 | | 0.86 |
| WMB | 0.30 | | 0.67 | | | 0.45 | | | 0.65 | | 0.86 |
| WMF x Prime | 0.59 | | 0.69 | | | 0.85 | | | 0.39 | | 0.86 |
| WMB x Prime | -0.46 | | 0.66 | | | -0.69 | | | 0.49 | | 0.86 |
|  |  | |  | | |  | | |  | |  |
| Younger adults |  | |  | | |  | | |  | |  |
| WMF | -0.34 | | 0.33 | | | -1.04 | | | 0.30 | | 0.86 |
| WMB | -0.41 | | 0.32 | | | -1.28 | | | 0.20 | | 0.86 |
| WMF x Prime | 0.32 | | 0.47 | | | 0.68 | | | 0.49 | | 0.86 |
| WMB x Prime | 0.63 | | 0.44 | | | 1.43 | | | 0.15 | | 0.86 |
| Dative sentences | | | | | | | | | | | |
|  | \| **β** \| **SE** \| ***z*** \| ***p*** \| \| --- \| --- \| --- \| --- \| | | | | | | | | | | ***R^2^*** |
| Older adults |  |  | | | | |  |  | | |  |
| WMF | -0.85 | 0.44 | | | | | -1.94 | 0.05 | | | 0.66 |
| WMB | -0.32 | 0.45 | | | | | -0.71 | 0.48 | | | 0.65 |
| WMF x Prime | -0.20 | 0.45 | | | | | -0.45 | 0.65 | | | 0.66 |
| WMB x Prime | 0.01 | 0.40 | | | | | 0.03 | 0.97 | | | 0.65 |
|  |  |  | | | | |  |  | | |  |
| Younger adults |  |  | | | | |  |  | | |  |
| WMF | 0.44 | 0.32 | | | | | 1.39 | 0.17 | | | 0.57 |
| WMB | 0.10 | 0.33 | | | | | 0.29 | 0.77 | | | 0.56 |
| WMF x Prime | -0.14 | 0.30 | | | | | -0.47 | 0.64 | | | 0.57 |
| WMB x Prime | 0.17 | 0.30 | | | | | 0.56 | 0.58 | | | 0.56 |

Supplementary Table 4

*Interactions between working memory scores and priming effects in eye fixations in the 0-400ms and 400-800ms time windows (WMF = Working memory forward score; WMB = Working memory backward score)*

|  | | Transitive sentences | | | | | | | | | | | | | | | | | | | | | | | | | | | | | | | | | | | | | | | | | | | | | | | | | | | | | | | | | | | | | | | | | | | | | | | | | | | | | | | | | | | | | | | | | | | | | | | | | | | | | | | | | | | | | | | | | | | | | | | | | | | |  |  |  |  |  |  |  |  |  |  |  |  |  |  |  |
| --- | --- | --- | --- | --- | --- | --- | --- | --- | --- | --- | --- | --- | --- | --- | --- | --- | --- | --- | --- | --- | --- | --- | --- | --- | --- | --- | --- | --- | --- | --- | --- | --- | --- | --- | --- | --- | --- | --- | --- | --- | --- | --- | --- | --- | --- | --- | --- | --- | --- | --- | --- | --- | --- | --- | --- | --- | --- | --- | --- | --- | --- | --- | --- | --- | --- | --- | --- | --- | --- | --- | --- | --- | --- | --- | --- | --- | --- | --- | --- | --- | --- | --- | --- | --- | --- | --- | --- | --- | --- | --- | --- | --- | --- | --- | --- | --- | --- | --- | --- | --- | --- | --- | --- | --- | --- | --- | --- | --- | --- | --- | --- | --- | --- | --- | --- | --- | --- | --- | --- | --- | --- | --- | --- | --- | --- | --- | --- | --- | --- | --- | --- | --- | --- | --- | --- | --- | --- | --- | --- | --- |
|  | | | | | | | | | | **0-400ms** | | | | | | | | | | | | | | | | | | | | | | | | | | | | | | | | | | | | | | | |  | | | | | | | | | | **400-800ms** | | | | | | | | | | | | | | | | | | | | | | | | | | | | | | | | | | | | | | | | | | | | | | | | | | | | | | | | | | | | | | | | |  | |  |  |  |  |  |  |  |  |  |  |  |  |  |  |
|  | | | | | | | | | | β | | | | | | SE | | | | | | | *z* | | | | | | | | | | | | *p* |  | | |  | | | | | | | | *R^2^* | | | | | | | | | | | | | | β | | | | | | |  | | | SE | | | | | | | | | | | | | | | | | | | | | | | *z* | *P* | | | | | | | | | | | | | | | | | | | | | *R^2^* | | | | | | | | | | | |  |  |  |  |  |  |  |  |  |  |  |  |  |
| Older adults | |  | | | | | | | | | | | | | | | |  | |  | | | | | | | | | | | | |  | | | | | | | | | | | | | | | | | | | | |  | | | | | | | | | | | | | | | |  | | | | | | | | | | | | | | | | | |  | | | | | | | | | | | | | | | | | | | | |  | | | | | | | | | | | | |  | | | | | | | | | | | | | | | |  |  |  |
| WMF | | | | | | | -0.18 | | | | | | | 0.14 | | | | | | | | | | | | -1.30 | | | | | | | | 0.19 | | | | 0.46 | | | | | | | | | | | | | | | | | | | | 0.10 | | | | | | | | | | | | | | | | | | 0.14 | | | | | | | | | | | | 0.70 | | | | | | | | | | | | | | | 0.48 | | | | | | |  | | | | | | | | | 0.31 | | | | | | | | |  |  |  |  |  |  |  |  |  |  |  |  |  |
| WMB | | | | | | | -0.14 | | | | | | | 0.14 | | | | | | | | | | | | -1.01 | | | | | | | 0.31 | | | | | | | | | | | | 0.46 | | | | | | | 0.22 | | | | | | | | | | | | | | | | | | | | 0.14 | | | | | | | | | | | 1.61 | | | | | | | | | | | | | 0.11 | | | | | | | | | | | | | | | | | | | | | | | 0.31 | | | | | | | | |  |  |  |  |  |  |  |  |  |  |  |  |  |
| WMF x Prime | | | | | | | 0.07 | | | | | | | 0.21 | | | | | | | | | | | | 0.38 | | | | | | | 0.71 | | | | | | | | | | | | 0.46 | | | | | | 0.06 | | | | | | | | | | | | | | | | | | | | | | | | | 0.21 | | | | | 0.29 | | | | | | | | | | | | | | | | | | 0.77 | | | | | | | | | | | 0.31 | | | | | | | | | | | | | | | | | | | | | | |  |  |  |  |  |  |  |  |
| WMB x Prime | | | | | | | 0.02 | | | | | | | 0.21 | | | | | | | | | | | | 0.10 | | | | | | | 0.91 | | | | | | | | | | | | 0.46 | | | | | | -0.14 | | | | | | | | | | | | | | | | | | | | | | | | | 0.21 | | | | | -0.69 | | | | | | | | | | | | | | | | | | 0.49 | | | | | | | | | | | 0.31 | | | | | | | | | | | | | | | | | | | | | | |  |  |  |  |  |  |  |  |
|  | | | | | | |  | | | | | | |  | | | | | | | | | | | |  | | | | | | |  | | | | | | | | | | | | | | | | | | | | | | | | | | | | |  | | | | |  | | | | | | | | | | | | |  | | | | | |  | | | | | | | | | | | | | | | | | | | | |  | | | | | | | | | | | | | | |  | | | | | | | | | | | | |  | | | | | |
| Younger adults | | | | | | |  | | | | | | |  | | | | | | | | | | | |  | | | | | | |  | | | | | | | | | | | | | | | | | | | | | | | | | | | | |  | | | | |  | | | | | | | | | | | | |  | | | | | |  | | | | | | | | | | | | | | | | | | | | |  | | | | | | | | | | | | | | |  | | | | | | | | | | | | |  | | | | | |
| WMF | | | | | | | -0.05 | | | | | | | 0.14 | | | | | | | | | | | | -0.36 | | | | | | | 0.72 | | | | | | | | | 0.34 | | | | | | | | | | | | | | | | 0.06 | | | | | | | | | | | | | | 0.14 | | | | | | | | | | | | | 0.39 | | | | | | | | | | | | | | | | | | 0.70 | | | | | | | | | | | | | | | | 0.20 | | | | | | | | |  |  |  |  |  |  |  |  |  |  |  |  |  |
| WMB | | | | | | -0.06 | | | | | | | 0.14 | | | | | | | | | | | | -0.41 | | | | | | | 0.68 | | | | | | | | 0.35 | | | | | | | | | | | | | | | | | | 0.05 | | | | | | | | | | | | | | 0.14 | | | | | | | | | | | | | 0.33 | | | | | | | | | | | | | | | | | | 0.74 | | | | | | | | | | | | | | | | 0.21 | | | | | | | | | | |  |  |  |  |  |  |  |  |  |  |  |
| WMF x Prime | | | | 0.26 | | | | | | | 0.21 | | | | | | | | | | | | | 1.21 | | | | | | 0.23 | | | | | | | | | | | | | | 0.34 | | | | | -0.11 | | | | | | | | | | | | | | | | | | | | | | | | 0.21 | | | | | | | | | -0.51 | | | | | | | | | | | | | | | | | | | | | | | | 0.61 | | | | | | | | 0.20 | | | | | | | | | | | | | | |  |  |  |  |  |  |  |  |  |  |  |  |
| WMB x Prime | | | | 0.36 | | | | | | | 0.20 | | | | | | | | | | | | | 1.78 | | | | | | 0.08 | | | | | | | | | | | | | | 0.35 | | | | | -0.38 | | | | | | | | | | | | | | | | | | | | | | | | 0.21 | | | | | | | | | -1.82 | | | | | | | | | | | | | | | | | | | | | | | 0.07 | | | | | | | | | | | | 0.21 | | | | | | | | | | | |  |  |  |  |  |  |  |  |  |  |  |  |
|  | Dative sentences | | | | | | | | | | | | | | | | | | | | | | | | | | | | | | | | | | | | | | | | | | | | | | | | | | | | | | | | | | | | | | | | | | | | | | | | | | | | | | | | | | | | | | | | | | | | | | | | | | | | | | | | | | | | | | | | | | | | | | | | | | |  |  |  |  |  |  |  |  |  |  |  |  |  |  |  |  |  |
|  | | | | | | | | | **0-400ms** | | | | | | | | | | | | | | | | | | | | | | | | | | | | | | | | | | | | | | |  | | | | | | | **400-800ms** | | | | | | | | | | | | | | | | | | | | | | | | | | | | | | | | | | | | | | | | | | | | | | | | | | | | | | | | | | | | | |  | | | | | |  |  |  |  |  |  |  |  |  |  |  |  |  |  |  |  |  |  |
|  | | | | | | | | β | | | | | | | SE | | | | | | *z* | | | | | | | | *p* | | | | | | | | | | *R2* | | | | | | | | |  | | | | | | | β | | | | | | | | | | | | | | | | | | | | | | | SE | | | | | | | | | | | *z* | | | | | | | | | | | | | *p* | | | | | | | | | | *R2* | | | | | | | | | | | | | | | | | | | |  |  |  |  |  |  |  |  |  |
| Older adults | | |  | | | | | | | | | | | | | |  | |  | | | | | | | | | |  | | | | | | | | | | | | | | | | | | |  | | | | | | | | | | | | | | | |  | | | | | | | | | | | | | | | | | | | | | | |  | | | | | | | | | |  | | | | | | | | | | | | | | | | | | | | | | | | | |  | | | | | | | | | | | | | | | |  |  |
| WMF | | | | -0.44 | | | | | | | 0.28 | | | | | | | | | | -1.59 | | | | | | 0.11 | | | | | | | | | | | | | | | | 0.71 | | | | | | | | | | | | | | | |  | | | | | | 0.13 | | | | | | | | | 0.26 | | | | | | | | | | | | | | | | 0.50 | | | | | | | | | | | | | | 0.62 | | | | | | | | |  | | | | | | | | 0.47 | | | | | | | |  |  |  |  |  |  |  |  |  |  |  |  |
| WMB | | | | -0.27 | | | | | | | 0.28 | | | | | | | | | | -0.99 | | | | | | 0.32 | | | | | | | | | | | | | | | | 0.70 | | | | | | | | | | | | | | | |  | | | | | | 0.40 | | | | | | | | | 0.26 | | | | | | | | | | | | | | | | 1.50 | | | | | | | | 0.14 | | | | | | | | | | | | | | | | | 0.46 | | | | | | | | | | | | | | | |  |  |  |  |  |  |  |  |  |  |
| WMF x Prime | | | | 0.87 | | | | | | | 0.28 | | | | | | | | | | 3.10 | | | | | | 0.002** | | | | | | | | | | | | | | | | 0.71 | | | | | | | | | | | | | | | |  | | | | | | -0.21 | | | | | | | | | 0.31 | | | | | | | | | | | | | | | | | | -0.69 | | | | | | 0.49 | | | | | | | | | | | | | | | | | 0.47 | | | | | | | | | | | | | | | | | | | | | |  |  |  |  |
| WMB x Prime | | | | 0.76 | | | | | | | 0.27 | | | | | | | | | | 2.77 | | | | | | | 0.006** | | | | | | | | | | | | | | | | | | 0.70 | | | | | | |  | | | | | | | | | | | | -0.72 | | | | | | | | | 0.32 | | | | | | | | | | | | | | | | | | -2.26 | | | | | | | | | 0.02* | | | | | | | | | | | | | | | | | 0.46 | | | | | | | | | | | | |  |  |  |  |  |  |  |  |  |  |
|  | | | |  | | | | | | |  | | | | | | | | | | | | |  | | | | | |  | | | | | | | | | | | | | | | | | | | | | | | | | | | | |  | | | | | |  | | | | | | | | | | | | | |  | | | | |  | | | | | | | | | | | | | | | | | | | | | | | |  | | | | | | | | | | | | | | |  | | | | | | | | | | | | |  | | | | |
| Younger adults | | | |  | | | | | | |  | | | | | | | | | | | | |  | | | | | |  | | | | | | | | | | | | | | | | | | | | | | | | | | | | |  | | | | | |  | | | | | | | | | | | | | |  | | | | |  | | | | | | | | | | | | | | | | | | | | | | | |  | | | | | | | | | | | | | | |  | | | | | | | | | | | | |  | | | | |
| WMF | | | | 0.42 | | | | | | | 0.19 | | | | | | | | | | -1.99 | | | | | | | | | 0.02* | | | | | | | | | 0.43 | | | | | | | | | | | | | | | | |  | | | | | | | | | 0.16 | | | | | | | | | 0.20 | | | | | | | | | | | | | | | | | 0.77 | | | | | | | | | | | | | 0.44 | | | | | | |  | | | | | | 0.12 | | | | | | | | | | | | | | |  |  |  |  |  |  |  |  |  |
| WMB | | | | | 0.38 | | | | | | | 0.20 | | | | | | | | | | 1.94 | | | | | | | | | 0.05 | | | | | | 0.43 | | | | | | | | | | | | | | | | | | | | | | | | | |  | | | | | | -0.5 | | | | | | 0.19 | | | | | | | | | | | | | | | | | | -0.24 | | | | | | | 0.81 | | | | | | | | | | |  | | | | | | | | | 0.13 | | | | | | | | | | | | | |  |  |  |  |  |  |  |
| WMF x Prime | | | | | -0.48 | | | | | | | 0.24 | | | | | | | | | | -1.98 | | | | | | | | | 0.05 | | | | | | | | | | 0.43 | | | | | | | | | | | | | | | |  | | | | | | | | | -0.39 | | | | | | | | | 0.27 | | | | | | | | | | | | | | | | | | -1.42 | | | | | | | 0.16 | | | | | | | | | | |  | | | | | | | | | 0.12 | | | | | | | | | | | | | |  |  |  |  |  |  |  |
| WMB x Prime | | | | | -0.15 | | | | | | | 0.26 | | | | | | | | | | -0.59 | | | | | | | | | 0.56 | | | | | | | | | | 0.43 | | | | | | | | | | | | | | | |  | | | | | | | | | -0.30 | | | | | | | | | | | 0.26 | | | | | | | | | | | | | | | | -1.14 | | | | | | | 0.25 | | | | | | | | | | | 0.13 | | | | | | | | | | | | | | | | | | | | | | | | | | | | | |
| Note: WMF = Working memory forward score; WMB = Working memory backward score | | | | | | | | | | | | | | | | | | | | | | | | | | | | | | | | | | | | | | | | | | | | | | | | | | | | | | | | | | | | | | | | | | | | | | | | | | | | | | | | | | | | | | | | | | | | | | | | | | | | | | | | | | | | | | | | | | | | | | | | | | | | | | | | | | | | | | | | | | | |  |
